# A meshless computational framework for studying cold spray additive manufacturing including large numbers of powder particles with diverse characteristics

**DOI:** 10.1038/s41598-024-62091-2

**Published:** 2024-05-18

**Authors:** Z. L. Zhang, M. Afrasiabi, M. Bambach

**Affiliations:** 1 Advanced Manufacturing Lab, ETH Zurich, Zürich, Switzerland; 2 Computational Manufacturing Group, inspire AG, Zürich, Switzerland

**Keywords:** Mechanical engineering, Computational science

## Abstract

Cold spray (CS) has emerged as an appealing additive manufacturing (AM) technique for producing or repairing individual components or entire structures. Compared to fusion-based AM technologies, cold spray additive manufacturing (CSAM) offers distinct advantages in the fabrication of components, while avoiding some melting/solidification-related issues such as phase transformation and oxidation. It involves intricate processes that pose significant challenges for numerical modeling, particularly when simulating the entire process at a large scale. The smoothed particle hydrodynamics (SPH) method is highly suitable for handling large material deformations due to its Lagrangian and meshless nature. In this work, we develop an enhanced SPH method to conduct large-scale simulations of CSAM with different powder sizes, morphologies, and distributions. A modified material model has been incorporated to accurately capture the strain-rate hardening effects during the plastic stage. The computational scale is greatly improved by using a Message Passing Interface (MPI) based framework, enabling the simulation of approximately ten million SPH particles. To the authors’ knowledge, this study marks the first attempt to numerically reproduce the entire process of CSAM with real powder sizes and distributions. Experimental data measured for a wide range of powder velocities are used to validate the simulation results and assess the prediction accuracy. Subsequently, we comparatively study the bonding mechanisms of powders with the same or different sizes, while also identifying a four-stage coating process. The effects of powder morphology on the bonding process are thoroughly investigated. A large-scale CSAM process is finally reproduced to demonstrate the capability of the present meshless scheme, and mechanisms of pore formation are analyzed, providing valuable insights for practical engineering applications.

## Introduction

Cold spray additive manufacturing (CSAM) is a process that uses compressed gas to propel powdered materials at high velocities, allowing them to be bonded and form solid objects without undergoing melting or heating^1^. The solid-state bonding mechanism distinguishes CSAM from the fusion-based AM techniques, such as laser powder bed fusion (LPBF)^2^ and direct energy deposition (DED)^3^. By avoiding melting of powders, CSAM effectively mitigates material oxidation, alteration of microstructure, and chemical degradation^4^. As a result, the CS technique attracts significant attention from both industry and academia, finding applications ranging from repair tasks and the fabrication of intricate free-standing components^5^.

Recent research has focused on using experimental or numerical approaches to understand the underlying mechanisms that dictate the final performance of CS products, as reviewed by Melentiev et al.^6^. However, there still exists no systematic understanding or explanation of the underlying forming and bonding mechanisms, particularly for the entire CS process. Bhattiprolu et al.^7^ carried out experiments to examine the effects of feedstock powder and processing parameters on properties of the microstructure during the CS bonding process. Seng et al.^8^ investigated the influences of spray angles on the surface roughness and microstructure thickness of coated structures based on experimental results. Bedard et al.^9^ experimentally studied the microstructure and micromechanical responses of CS with Al-6061 powders. Different approaches, such as atomized and heat-treated techniques, were adopted by them to perform analyses on the individual slats and evaluate the mechanical behaviors of the materials. Chen et al.^10^ discovered that the hot isostatic pressing treatment was an effective tool for reducing interior structural defects, adjusting the microstructure, and thus improving mechanical properties. In addition to the bonding phenomenon and mechanical property, the entire CS system and the influences of processing parameters have also been investigated by researchers^11^. In CSAM, the extremely high powder strain rates in the order of 10^7^ to 10^9^ s^−1^ make it nearly impossible to observe the deposition process using conventional experimental tools^4^. The temporal evolutions of powder deformation, temperature, stress, and microstructure are very difficult to capture experimentally due to the limitations of the equipment in resolving micron-sized powders.

Numerical modeling has proven effective in providing the missing information and intricate details during the CSAM process^12^. In the early stages, the purely Lagrangian finite element method (FEM) was often applied to study CSAM. For instance, Kim et al.^13^ conducted FEM simulations to analyze the influences of interface oxygen on the stress and temperature surrounding the aluminum coating interfaces. Ghelichi et al.^14^ employed FEM to determine the critical velocity of a powder required to achieve effective coating with the substrate. To address the issue of element distortion resulting from the substantial deformation of powders, the Eulerian FEM scheme has been utilized, where two overlapping meshes consisting of a background spatial mesh were adopted^15^. Moreover, to leverage the advantages of both Lagrangian and Eulerian descriptions, some coupled schemes were developed for CSAM modeling^5^. The Arbitrary Lagrangian–Eulerian (ALE) approach was applied by Pan et al.^16^ to analyze the impact and bonding processes of a single powder, where the influencing factors of impact velocity and power temperature were evaluated. Lordejani et al.^17^ employed the Coupled Eulerian Lagrangian (CEL) method to assess the role of powder features on the quality of CS deposits. Despite some successful applications of FEM analyses on CSAM, accurately and efficiently reproducing the extremely large deformations of powders, including jetting formation and interfacial bonding, remains a significant challenge.

To overcome the barriers in mesh-based modeling, the smoothed hydrodynamics method (SPH)^18,19^, a Lagrangian and particle-based method, has been introduced to reproduce the deposition process of CS and adequately resolve material plastic flows. SPH has unique advantages in modeling the fusion-based AM and solid-state metal machining^20–22^, i.e., avoiding mesh distortions and naturally capturing interfaces. Russell et al.^23^, Fürstenau et al.^24^, Lüthi et al.^25^, and Meier et al.^26^ respectively proposed various SPH schemes to simulate and study the processes of laser powder bed fusion. In the context of solid-state CSAM problems, SPH shows its great effectiveness in capturing the large deformations of metal powders. Hemeda et al.^27^ developed an SPH method to simulate the single powder CS process with the consideration of oxide layer effects. Li et al.^28^ numerically studied the influences of impact angle on powder deformations in CS using the SPH method. Zhang et al.^29,30^ developed a density adaptive SPH method to deal with the solid-state forming and welding problems. Moreover, they incorporated several correction techniques into SPH to simulate CSAM involving multiple powders^31^. However, only a small number of powders with a uniform powder size were adopted in their simulations based on a conventional material model.

To study the important underlying mechanisms of CSAM, multi-powder impact models have been adopted, which considered fundamental aspects of the CS process and enables researchers to examine interactions between multiple powders. These complex mechanisms are usually not accessible with the use of a single-powder impact model. Wang et al.^32^ conducted a numerical analysis of residual stress in CS with multiple spherical powders arranged in a staggered pattern. Msolli et al.^33^ employed a multi-powder FEM model to study the microstructure evolution in the CS process. In their model, powders of the same size were uniformly distributed and simultaneously impacted onto the substrate. Gao et al.^34^ applied the molecular dynamics (MD) method to investigate the tamping effect in CSAM with copper powders, once again assuming regular distribution and uniform size among the powders. In the above and some other three-dimensional (3D) numerical studies, the CSAM process was generally oversimplified in the following aspects: (1) all powders were assumed to be spheres of the same size, (2) the powders were arranged on a regular grid or in a staggered pattern, (3) only a small number (typically fewer than 100) of powders were analyzed. In view of such limitations of existing research, large-scale simulations and analyses with the consideration of realistic CS parameters (e.g., powder size, distribution, and morphology) are deemed necessary for a better understanding and more insightful knowledge of the process.

In numerical modeling of the CSAM process, material models play a pivotal role in overall reproducibility as they directly influence accuracy of the predicted material deformations. Up to now, the Johnson–Cook (JC) model serves as the prevalent choice in computational investigations of CSAM due to its ability to capture metal plastic flows at high strain rates^5,35^. However, in the conventional JC model, the flow stress exhibits a linear dependence on the strain rate. The strain-rate hardening effects or the strain rate sensitivity at higher strain rates cannot be effectively captured, which may cause overlarge or exaggerated material deformations, as observed by Weiller and Delloro^36^. In view of this, some modifications to the JC material model have been reported. Tuazon et al.^37^ introduced a logarithmic power-law model by including additional material constants in the description of strain rate sensitivity. However, this model may introduce nonphysical stresses at low strain rates. El-Qoubaa and Othman^38^ proposed a modified Eyring model based on the original JC model, which yielded results close to experimental data. Nevertheless, their model includes a lot of material-dependent constants. In practice, CSAM applications often encounter strain rates that surpass the upper limit of effectiveness for the JC model of various materials, such as aluminum (Al). Therefore, an effective material model that can capture the strain rate sensitivity is of paramount importance to understand the mechanics of CSAM processes.

The objective of this work is to implement 3D large-scale modeling of the complete CSAM process by incorporating actual powder sizes, morphologies, and distributions. Experimental validations are conducted to demonstrate accuracy of the developed model. Furthermore, the underlying coating mechanisms regarding multiple powder scenarios are examined. As for the CSAM modeling, most existing research focused on studying the coating or bonding mechanisms of a single powder^4,27^ or powders arranged in regular grids or staggered patterns^33^. The maximum number of powders considered in high-fidelity simulations from other sources is usually in the magnitude of hundreds^36,39^. In contrast, our investigation introduces an enhanced SPH solver based on MPI parallel computing, which can handle a significantly larger number of powders (exceeding ten thousand) within realistic CSAM process conditions. The large deformations of materials can be well captured by considering the effects of strain rate sensitivity in the JC model. Our meshless computational framework carries the following important features: a decoupled finite particle method, a modified constitutive model, enabling parallel computations, an average velocity model, and the adaptive smoothing length model. The powders are generated using the discrete element method (DEM) based on experimentally reported sizes and positions. This enables investigation of coating mechanisms in a real-parameter model set-up, which has been rarely reported, if not unprecedented. The present meshless scheme exhibits significant potential in the modeling and enhancing our understanding of large-scale CSAM problems.

This paper is structured as follows. The fundamental concepts of SPH and some improved techniques are first introduced. The modified constitutive model, the equation of state, and the DEM model are described in detail. Furthermore, we simulate the single powder CS with numerical results compared to the experimental data. Subsequently, the entire process of CS with different powder sizes, morphologies and distributions are modeled and investigated. Finally, a few concluding remarks are given.

## Methodology

### Basic concepts of SPH

In SPH, two steps are employed to discretize the governing partial differential equations, i.e., kernel and particle approximations^40,41^. The kernel approximation uses the following integral equations to approximate the field variable *f* (**x**) and its spatial derivative at a spatial position **x**,

1 $$\left\langle {f({\mathbf{x}})} \right\rangle = \int\limits_{\Omega } {f({\mathbf{x}}{\prime} )} W({\mathbf{x}} - {\mathbf{x}}{\prime} ,h){\text{d}}{\mathbf{x}}{\prime} {\mathbf{,}}$$

2 $$\left\langle {\nabla \cdot f({\mathbf{x}})} \right\rangle = - \int\limits_{\Omega } {f({\mathbf{x}}{\prime} )\nabla W({\mathbf{x}} - {\mathbf{x}}{\prime} ,h)} {\text{d}}{\mathbf{x}}{\prime} {\mathbf{,}}$$

where <  > represents the SPH approximation and will be dropped in the following equations. The kernel function is denoted as *W*, Ω is the support or influence domain, and the smoothing length is *h*. Subsequently, the particle approximation is applied to compute the field variable of a particle *i*, which can be written as

3 $$f({\mathbf{x}}_{i} ) \approx \sum\limits_{j = 1}^{N} {\frac{{m_{j} }}{{\rho_{j} }}f({\mathbf{x}}_{j} )} W({\mathbf{x}}_{j} - {\mathbf{x}}{}_{i}),$$

4 $$\nabla \cdot f({\mathbf{x}}_{i} ) \approx \sum\limits_{j = 1}^{N} {\frac{{m_{j} }}{{\rho_{j} }}} f({\mathbf{x}}_{j} )\nabla_{i} W_{ij} ,$$

where *m*_*j*_ and *ρ*_*j*_ show the mass and density of a neighboring particle *j*. The summation is calculated with regard to all neighboring particles of the concerned one, with *N* denoting the total number of neighboring particles.

### Discretization of governing equations

For modeling large solid deformations, the governing equations of continuum mechanics can be written as

5 $$\left\{ \begin{gathered} \frac{{{\text{d}}\rho }}{{{\text{d}}t}} = - \rho \nabla \cdot {\mathbf{v}} \hfill \\ \frac{{{\text{d}}{\mathbf{v}}}}{{{\text{d}}t}} = \frac{1}{\rho }\nabla \cdot {{\varvec{\upsigma}}} \hfill \\ \frac{{{\text{d}}e}}{{{\text{d}}t}} = \frac{1}{\rho }{{\varvec{\upsigma}}}:\nabla {\mathbf{v}} - \frac{K}{\rho }\nabla^{2} T, \hfill \\ \end{gathered} \right.$$

where **v**, *ρ*, *t*, σ, *e*, *K* and *T* denote the velocity, density, time, stress, internal energy, thermal conductivity, and temperature, respectively.

By considering the SPH approximations, the discretized forms of the governing equations are given by

6 $$\left\{ \begin{gathered} \frac{{{\text{d}}\rho_{i} }}{{{\text{d}}t}} = \rho_{i} \sum\nolimits_{j = 1}^{N} {\frac{{m_{j} }}{{\rho_{i} }}\left( {v_{i}^{\beta } - v_{j}^{\beta } } \right)\frac{{\partial W_{ij} }}{{\partial x_{i}^{\beta } }}} \hfill \\ \frac{{{\text{d}}v_{i}^{\alpha } }}{{{\text{d}}t}} = - \sum\nolimits_{j = 1}^{N} {m_{j} \left( {\frac{{\sigma_{i}^{\alpha \beta } }}{{\rho_{i}^{2} }} + \frac{{\sigma_{j}^{\alpha \beta } }}{{\rho_{j}^{2} }} + \Pi_{ij} } \right)\frac{{\partial W_{ij} }}{{\partial x_{i}^{\beta } }}} \hfill \\ \frac{{{\text{d}}e_{i} }}{{{\text{d}}t}} = \frac{1}{2}\sum\nolimits_{j = 1}^{N} {m_{j} \left( {\frac{{P_{i} }}{{\rho_{i}^{2} }} + \frac{{P_{j} }}{{\rho_{j}^{2} }} + \Pi_{ij} } \right)\left( {v_{i}^{\beta } - v_{j}^{\beta } } \right)\frac{{\partial W_{ij} }}{{\partial x_{i}^{\beta } }} + \frac{1}{{\rho_{i} }}S_{i}^{\alpha \beta } \varepsilon_{i}^{\alpha \beta } } \hfill \\ \frac{{{\text{d}}x_{i}^{\alpha } }}{{{\text{d}}t}} = v_{i}^{\alpha } , \hfill \\ \end{gathered} \right.$$

where *α* and *β* are tensor indices varying from 1 to the dimension of the problem. *P* is the pressure, *S* represents deviatoric part of the stress, and *ε* shows the strain. Π stands for the artificial viscosity that is used to alleviate numerical instabilities induced by the discontinuity of shockwaves^19^.

### Decoupled finite particle method (DFPM)

In this work, we extend the decoupled finite particle method (DFPM) to model a manufacturing problem for the first time. It is known that the conventional SPH is limited by its first-order accuracy. Within the process and manufacturing domain, the majority of existing numerical simulation developments are based on the original formulation of SPH. In our previous work, we developed a DFPM approach^42,43^ to improve the accuracy of SPH while ensuring its flexibility and efficiency. Since the creation of DFPM, many researchers have extended its application to simulate various nonlinear fluid and solid dynamics problems, such as fluid flows in porous media^44^, composite materials impacting problem^45^, and transient viscoelastic flows^46^. In this work, we use DFPM for modeling CSAM in the following way:

Based on the Taylor series expansion at a neighboring point **x**_*i*_ of **x**, the function value can be obtained as

7 $$f({\mathbf{x}}) = f_{i} + f_{i,\alpha } (x^{\alpha } - x_{i}^{\alpha } ) + r(({\mathbf{x}} - {\mathbf{x}}_{i} )^{2} ),$$

where $f_{i}$ and $f_{i,\alpha }$ are

8 $$f_{i} = f({\mathbf{x}}_{i} ),$$

9 $$f_{i,\alpha } = (\partial f/\partial x_{{}}^{\alpha } )_{i} .$$

By multiply a kernel function $W({\mathbf{x}} - {\mathbf{x}}_{i} )$ to both sides of Eq. (7) and performing the integration over the supporting domain, we obtain

10 $$\int\limits_{\Omega } {f({\mathbf{x}})W({\mathbf{x}} - {\mathbf{x}}_{i} )} {\text{d}}{\mathbf{x}} = f_{i} \int\limits_{\Omega } {W({\mathbf{x}} - {\mathbf{x}}_{i} )} {\text{d}}{\mathbf{x}} + \nabla f_{i} \int\limits_{\Omega } {({\mathbf{x}} - {\mathbf{x}}_{i} )W({\mathbf{x}} - {\mathbf{x}}_{i} )} {\text{d}}{\mathbf{x}},$$

where Ω will be dropped in the following equations for the sake of conciseness. Likewise, using the kernel derivative $\nabla W({\mathbf{x}}_{i} )$ to replace the kernel function in Eq. (10), we further attain the following equation,

11 $$\int {f({\mathbf{x}})\nabla W({\mathbf{x}} - {\mathbf{x}}_{i} )} {\text{d}}{\mathbf{x}} = f_{i} \int {\nabla W({\mathbf{x}} - {\mathbf{x}}_{i} )} {\text{d}}{\mathbf{x}} + \nabla f_{i} \int {({\mathbf{x}} - {\mathbf{x}}_{i} )} \nabla W({\mathbf{x}} - {\mathbf{x}}_{i} ){\text{d}}{\mathbf{x}}{\mathbf{.}}$$

Based on Eqs. (10) and (11), the matrix equation is obtained:

12 $$\left[ \begin{gathered} \int {W({\mathbf{x}} - {\mathbf{x}}_{i} )} {\text{d}}{\mathbf{x}}\int {({\mathbf{x}} - {\mathbf{x}}_{i} )} W({\mathbf{x}} - {\mathbf{x}}_{i} ){\text{d}}{\mathbf{x}} \hfill \\ \int {\nabla W({\mathbf{x}} - {\mathbf{x}}_{i} )} {\text{d}}{\mathbf{x}}\int {({\mathbf{x}} - {\mathbf{x}}_{i} )} \nabla W({\mathbf{x}} - {\mathbf{x}}_{i} ){\text{d}}{\mathbf{x}} \hfill \\ \end{gathered} \right]\left\{ \begin{gathered} f_{i} \hfill \\ \nabla f_{i} \hfill \\ \end{gathered} \right\} = \left[ \begin{gathered} \int {f({\mathbf{x}})W({\mathbf{x}} - {\mathbf{x}}_{i} )} {\text{d}}{\mathbf{x}} \hfill \\ \int {f({\mathbf{x}})\nabla W({\mathbf{x}} - {\mathbf{x}}_{i} )} {\text{d}}{\mathbf{x}} \hfill \\ \end{gathered} \right].$$

Solving for the field variable *f* and its gradient yields

13 $$\left\{ \begin{gathered} f_{i} \hfill \\ \nabla f_{i} \hfill \\ \end{gathered} \right\} = {\mathbf{L}}^{ - 1} \left[ \begin{gathered} \int {f({\mathbf{x}})W({\mathbf{x}} - {\mathbf{x}}_{i} )} {\text{d}}{\mathbf{x}} \hfill \\ \int {f({\mathbf{x}})\nabla W({\mathbf{x}} - {\mathbf{x}}_{i} )} {\text{d}}{\mathbf{x}} \hfill \\ \end{gathered} \right],$$

where

14 $${\mathbf{L}} = \left[ \begin{gathered} \int {W({\mathbf{x}} - {\mathbf{x}}_{i} )} {\text{d}}{\mathbf{x}}\int {({\mathbf{x}} - {\mathbf{x}}_{i} )} W({\mathbf{x}} - {\mathbf{x}}_{i} ){\text{d}}{\mathbf{x}} \hfill \\ \int {\nabla W({\mathbf{x}} - {\mathbf{x}}_{i} )} {\text{d}}{\mathbf{x}}\int {({\mathbf{x}} - {\mathbf{x}}_{i} )} \nabla W({\mathbf{x}} - {\mathbf{x}}_{i} ){\text{d}}{\mathbf{x}} \hfill \\ \end{gathered} \right].$$

This matrix equation based on finite particle method (FPM) keeps second order accuracy of the solution as the first order derivatives are retained.

Due to the matrix equation involved, FPM is computationally expensive. More importantly, the corrective matrix can be ill-conditioned in practical simulations, which decreases computational stability. To improve computational efficiency and stability, the primary contribution of the corrective matrix **L** is considered to decouple the matrix equation. Specifically, the contribution from the self-direction of a function is dominant in estimating its derivative, while contributions from other directions are ignored. This idea is inspired by the finite difference approach, where the derivative in a certain direction is often substituted with the finite difference along that direction. Therefore, by treating the main diagonal terms in **L** as dominant and ignoring other terms, we obtain

15 $${\mathbf{L^{\prime}}} = \left( {\begin{array}{*{20}c} {\int {W{\text{d}}V} } & {0} & {0} & {0} \\ {0} & {\int {(x - x_{i} )} W_{x}^{\prime } {\text{d}}V} & {0} & {0} \\ {0} & {0} & {\int {(y - y_{i} )} W_{y}^{\prime } {\text{d}}V} & {0} \\ {0} & {0} & {0} & {\int {(z - z_{i} )} W_{z}^{\prime } {\text{d}}V} \\ \end{array} } \right).$$

The new matrix ${\mathbf{L^{\prime}}}$ effectively decouples the computation of a field variable *f* and its derivatives. In this method, *f* and its derivatives are expressed by the particle approximation as

16 $$\left\{ \begin{gathered} f_{i} = \frac{{\sum\nolimits_{j = 1}^{N} {f_{j} W_{ij} } \Delta V_{j} }}{{\sum\nolimits_{j = 1}^{N} {W_{ij} } \Delta V_{j} }} \hfill \\ f_{i,x} = \frac{{\sum\nolimits_{j = 1}^{N} {f_{j} \frac{{\partial W_{ij} }}{{\partial x_{i} }}} \Delta V_{j} }}{{\sum\nolimits_{j = 1}^{N} {x_{ji} \frac{{\partial W_{ij} }}{{\partial x_{i} }}} \Delta V_{j} }} \hfill \\ f_{i,y} = \frac{{\sum\nolimits_{j = 1}^{N} {f_{j} \frac{{\partial W_{ij} }}{{\partial y_{i} }}} \Delta V_{j} }}{{\sum\nolimits_{j = 1}^{N} {y_{ji} \frac{{\partial W_{ij} }}{{\partial y_{i} }}} \Delta V_{j} }} \hfill \\ f_{i,z} = \frac{{\sum\nolimits_{j = 1}^{N} {f_{j} \frac{{\partial W_{ij} }}{{\partial z_{i} }}} \Delta V_{j} }}{{\sum\nolimits_{j = 1}^{N} {z_{ji} \frac{{\partial W_{ij} }}{{\partial z_{i} }}} \Delta V_{j} }}, \hfill \\ \end{gathered} \right.$$

where *V* represents the volume of a particle. In practical problems, the asymmetric form of Eq. (16) can also be used,

17 $$\left\{ \begin{gathered} f_{i} = \frac{{\sum\nolimits_{j = 1}^{N} {f_{j} W_{ij} } \Delta V_{j} }}{{\sum\nolimits_{j = 1}^{N} {W_{ij} } \Delta V_{j} }} \hfill \\ f_{i,x} = \frac{{\sum\nolimits_{j = 1}^{N} {(f_{j} - f_{i} )\frac{{\partial W_{ij} }}{{\partial x_{i} }}} \Delta V_{j} }}{{\sum\nolimits_{j = 1}^{N} {x_{ji} \frac{{\partial W_{ij} }}{{\partial x_{i} }}} \Delta V_{j} }} \hfill \\ f_{i,y} = \frac{{\sum\nolimits_{j = 1}^{N} {(f_{j} - f_{i} )\frac{{\partial W_{ij} }}{{\partial y_{i} }}} \Delta V_{j} }}{{\sum\nolimits_{j = 1}^{N} {y_{ji} \frac{{\partial W_{ij} }}{{\partial y_{i} }}} \Delta V_{j} }} \hfill \\ f_{i,z} = \frac{{\sum\nolimits_{j = 1}^{N} {(f_{j} - f_{i} )\frac{{\partial W_{ij} }}{{\partial z_{i} }}} \Delta V_{j} }}{{\sum\nolimits_{j = 1}^{N} {z_{ji} \frac{{\partial W_{ij} }}{{\partial z_{i} }}} \Delta V_{j} }}. \hfill \\ \end{gathered} \right.$$

During the CSAM process, phase changes and jet formation commonly occur. It is very difficult to apply FPM to model such a strongly nonlinear problem with large material deformations. This is why FPM has been rarely employed in impact dynamics simulations, despite its success in various fluid dynamics problems. In comparison, DFPM appears very suitable to simulate large solid deformations as it does not require a matrix equation. This feature makes it highly attractive for particle modeling of CSAM problems.

### XSPH model

In large material deformations, the problem of tensile instability often arises, which can result in clustering of particles or formation of voids. To solve this problem, we employ the XSPH model proposed by Gray et al.^47^. The average velocity of the neighboring particles can be used to modify the local velocity of a particle, which is written as

18 $${\overline{\mathbf{v}}}_{i} = {\mathbf{v}}_{i} + \varsigma \sum\limits_{j = 1}^{N} {\frac{{m_{j} }}{{\rho_{i} }}{\mathbf{v}}_{ij} W_{ij} } ,$$

where the parameter *ς* is usually set as 0.5. The kernel function *W*_*ij*_, rather than the kernel gradient value, is selected for the correction. It is noted that the conservation of momentum is maintained when using the correction, as **v**_*ji*_ remains the negative component of the term **v**_*ij*_ with respect to particle *j*. The correction velocity can be obtained in different ways, and XSPH has been demonstrated to be effective for modeling the solid behaviors.

### Adaptive smoothing length

To ensure better numerical stability, especially in the presence of large deformations of the powders in CSAM, we utilize an adaptive smoothing length scheme that updates the SPH kernel support according to the variations in particle density. In solid impact problems, a larger value for the smoothing length should be adopted compared to the one used in fluid flow modeling. However, choosing a larger smoothing length can decrease computational efficiency due to the increased number of interaction pairs. Additionally, irrelevant information of particles may also be included in the simulations. In contrast, when a smaller smoothing length is applied, approximating field variables of the concerned particle is difficult since information from neighboring particles is insufficient. For CSAM problems, materials experience large deformations and plastic flow, leading to instances of extremely scattered or gathered particles. This problem should be carefully handled as it can affect computational accuracy and stability. Therefore, we employ a variable smoothing length determined by the rate of variations in density of the concerned particle^48^,

19 $$\frac{{{\text{d}}h_{i} }}{{{\text{d}}t}} = - \frac{1}{d}\frac{{h_{i} }}{{\rho_{i} }}\frac{{{\text{d}}\rho_{i} }}{{{\text{d}}t}},$$

where *d* is the dimension of the problem. In this way, the number of neighboring particles can be adaptively adjusted so that the information of the concerned particle can be appropriately calculated.

## Numerical models

### Modified constitutive model

The stress tensor in Eq. (5) can be decomposed into a spherical isotropic part and a residual deviatoric part

20 $$\sigma^{\alpha \beta } = - P\delta^{\alpha \beta } + S^{\alpha \beta } ,$$

where *δ*^*αβ*^ represents the component of a unit tensor. *P* and *S*^*αβ*^ are obtained by the equation of state and constitutive model, respectively. The Jaumann stress rate is usually employed to compute the temporal evolution of the deviatoric stress,

21 $$\dot{S}_{J}^{\alpha \beta } = \dot{S}^{\alpha \beta } - S^{\alpha \eta } \dot{\omega }^{\beta \eta } - \dot{\omega }^{\beta \eta } S^{\eta \beta } ,$$

where $\dot{\omega }$ is the rotation tensor. The constitutive relationship in the elastic stage is given by

22 $$\dot{S}_{J}^{\alpha \beta } = 2G\left( {\dot{\varepsilon }^{\alpha \beta } - \frac{1}{3}\delta^{\alpha \beta } \dot{\varepsilon }^{\alpha \alpha } } \right).$$

*G* and $\dot{\varepsilon }^{\alpha \alpha }$ are the shear modulus and the volumetric strain rate, respectively. Moreover, the elastic deviatoric stress rate is written as

23 $$\dot{S}_{e}^{\alpha \beta } = S^{\alpha \eta } \dot{\omega }^{\beta \eta } + S^{\eta \beta } \dot{\omega }^{\alpha \eta } - 2G\left( {\dot{\varepsilon }^{\alpha \beta } - \frac{1}{3}\delta^{\alpha \beta } \dot{\varepsilon }^{\alpha \alpha } } \right),$$

where *η* stands for a tensor index. The strain rate and rotation tensors in above equations are given by

24 $$\left\{ \begin{gathered} \dot{\varepsilon }^{\alpha \beta } = \frac{1}{2}\left( {\frac{{\partial v^{\alpha } }}{{\partial x^{\beta } }} + \frac{{\partial v^{\beta } }}{{\partial x^{\alpha } }}} \right) \hfill \\ \dot{\omega }^{\alpha \beta } = \frac{1}{2}\left( {\frac{{\partial v^{\alpha } }}{{\partial x^{\beta } }} - \frac{{\partial v^{\beta } }}{{\partial x^{\alpha } }}} \right). \hfill \\ \end{gathered} \right.$$

For metals and alloys, some split-Hopkinson bar experiments showed that the material strain-state hardening might dramatically increase at a higher strain rate, e.g., 10^3^ s^−1^ reported for Al 6061^49^. CSAM is known to exhibit strain rates usually larger than 10^3^ s^−1^. To describe the plastic behavior of such materials at very high strain rates, a modified Johnson–Cook model^49^ is adopted. In this model, the yield stress is computed as

25 $$\sigma_{Y} = (A + B\varepsilon_{p}^{n} )\left( {1 + C\ln \left( {\frac{{\dot{\varepsilon }_{p} }}{{\dot{\varepsilon }_{0} }}} \right)} \right)\left[ {1 - \left( {\frac{{T - T_{room} }}{{T_{melt} - T_{room} }}} \right)^{m} } \right],$$

where

26 $$C = \left\{ \begin{gathered} C_{1} \,\,\,{\text{and}}\,\,\,\dot{\varepsilon }_{0} = 1,\,\,{\text{if}}\,\,\dot{\varepsilon }_{p} < \dot{\varepsilon }_{c} \hfill \\ C_{2} \,\,\,{\text{and}}\,\,\,\dot{\varepsilon }_{0} = \dot{\varepsilon }_{c} ,\,\,\,{\text{if}}\,\,\dot{\varepsilon }_{p} > \dot{\varepsilon }_{c} \hfill \\ \end{gathered} \right.\,\,\,\,\,\,{\text{with}}\,\,C_{2} > C_{1}.$$

$T_{room}$ and $T_{melt}$ denote the room and melting temperature, respectively. $\varepsilon_{p}$ represents the effective plastic strain, and $\dot{\varepsilon }_{c}$ is the critical plastic strain rate. *A*, *B*, and the superscripts *n* and *m* in Eq. (25) are material constants obtained from experiments. *C*_1_ and* C*_2_ represent the increasing coefficients in the yield stress when the effective plastic strain rate is higher than a critical value. In this way, the increase of strain rate hardening is considered, so that extremely high strain rate of metal materials can be effectively predicted.

With the yield stress computed by the modified JC model, the deviatoric stress in the plastic stage is obtained as

27 $$S^{\alpha \beta } = \sqrt {\frac{{\sigma_{Y}^{2} }}{3J}} S_{e}^{\alpha \beta } ,J > \sigma_{Y}^{2} /3.$$

Moreover, we need to update the temperature based on the internal energy variation d*e*, which is given by

28 $$T^{(n + 1)} = T^{(n)} + \frac{{{\text{d}}e}}{{mC_{V} }},$$

where $C_{V}$ is the specific heat. $T^{(n)}$ and $T^{(n + 1)}$ are the temperature values computed at the corresponding time steps. When the material deforms plastically, the incremental plastic work $\Delta W_{p}^{\left( n \right)}$ should be calculated for the update of temperature,

29 $$\left\{ \begin{gathered} \Delta W_{p}^{\left( n \right)} = \frac{1}{2}\left( {\sigma_{eff}^{{\left( {n + 1} \right)}} + \sigma_{eff}^{\left( n \right)} } \right)\Delta \varepsilon_{eff}^{\left( n \right)} \left( {\frac{m}{{\rho^{{\left( {n + \frac{1}{2}} \right)}} }}} \right) \hfill \\ \sigma_{eff}^{{\left( {n + 1} \right)}} = \sqrt {\frac{3}{2}{\text{S}}_{e}^{{\left( {n + 1} \right)}} :{\text{S}}_{e}^{{\left( {n + 1} \right)}} } \hfill \\ \hfill \\ \Delta \varepsilon_{eff}^{\left( n \right)} = \frac{{\sigma_{eff}^{\left( n \right)} - \sigma_{Y} }}{3G}, \hfill \\ \end{gathered} \right.$$

where $\sigma_{eff}$, $\varepsilon_{eff}$ and $\Delta \varepsilon_{eff}^{\left( n \right)}$ stand for effective plastic stress, effective plastic strain, and effective plastic strain increment at the corresponding time step, respectively. $\rho_{{}}^{{\left( {n{ + }\frac{1}{2}} \right)}}$ is the value of density at the half time step.

### Equation of state

In CSAM, high pressure and adiabatic heating may result in phase changes of metal materials in local regions. To consider the phase change and possible gasification effects of the Al material, the Tillotson equation of state (EOS)^50^ is employed. In this EOS, pressure of the material is divided into four stages as solid, liquid, vapor–liquid mixture, and vapor (*p*_1_ to *p*_4_),

30 $$\left\{ \begin{gathered} p_{1} = \left( {a + \frac{b}{{w_{0} }}} \right)\eta \rho_{0} e + A_{1} \mu + B_{1} \mu^{2} \hfill \\ p_{2} = \left( {a + \frac{b}{{w_{0} }}} \right)\eta \rho_{0} e + A_{1} \mu \hfill \\ p_{3} = p_{2} + \frac{{(p_{4} - p_{2} )(e - e_{s} )}}{{(e_{s}{\prime} - e_{s} )}} \hfill \\ p_{4} = a\eta \rho_{0} e + \left( {\frac{{b\eta \rho_{0} e}}{{w_{0} }} + A_{1} \mu e^{\xi x} } \right)e^{{ - \lambda x^{2} }} , \hfill \\ \end{gathered} \right.$$

where

31 $$\eta = \frac{\rho }{{\rho_{0} }},\mu = \eta - 1,w_{0} = 1 + \frac{e}{{e_{0} \eta^{2} }},$$

*A*_1_, *B*_1_,* a*, *b*, *λ*, *ξ*, *e*_0_, *e*_*s*_ and *e*^’^ are experimentally determined material parameters. The specific parameters for Al-6061 and sapphire used in the constitutive modeling and equation of state are listed in Table 1.

**Table 1:** Material properties of Al-6061 and sapphire for the present simulations^51^.

Property	Parameter	Al-6061	Sapphire
General	Density (kg/m^3^)	2700	3980
	Thermal conductivity (W m^−1^ K^−1^)	154	33
	Specific heat (J kg^−1^ K^−1^)	1009	755
	*T*_*room*_ (K)	298	298
	*T*_*melt*_ (K)	925	–
Elastic	Young’s modulus (GPa)	69	416
	Poisson’s ratio	0.331	0.231
Plastic	*A* (MPa)	270	–
	*B* (MPa)	154.3	–
	*n*	0.239	–
	*m*	1.42	–
	*C*_1_	0.002	–
	*C*_2_	0.029	–
	Reference strain rate (s^−1^)	1	–
	$\dot{\varepsilon }_{c}$(s^−1^)	597	–
EOS	*A*_1_ (MPa)	75.2	–
	*B*_1_ (MPa)	65	–
	*a*	0.5	–
	*b*	1.63	–
	*λ*	5.0	–
	*ξ*	5.0	–
	*e*_*s*_ (kJ g^−1^)	3.0	–
	*e*_0_ (kJ g^−1^)	5.0	–
	*e*^’^ (kJ g^−1^)	15.0	–

### DEM model for powder generation

**Figure 1:**
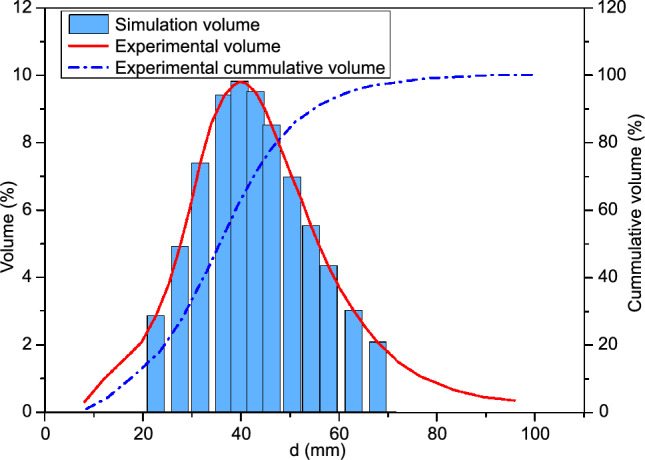
Powder size distribution observed by experiments^52^ (curve) and used for simulation (histogram).

In this section, the DEM model is introduced that is used to generate the powders. To study the bonding effects of multiple powders with different sizes, we select the volume fraction of Al-6061 powders obtained from experiments^52^ for the current numerical simulations. As depicted in Fig. 1, the experimentally obtained powder size distribution curve is represented by bar histograms to generate powders of varying sizes. There are very few powders with diameters smaller than 20 µm or larger than 70 µm. Consequently, powders with extremely small or large sizes are not considered in the present simulations. In CSAM operations, the morphology of the powder has significant effects on both the impact velocity and the deposition quality^53^. Therefore, we also generate ellipsoidal powders, which are commonly observed, to examine the influences of powder morphology.

**Figure 2:**
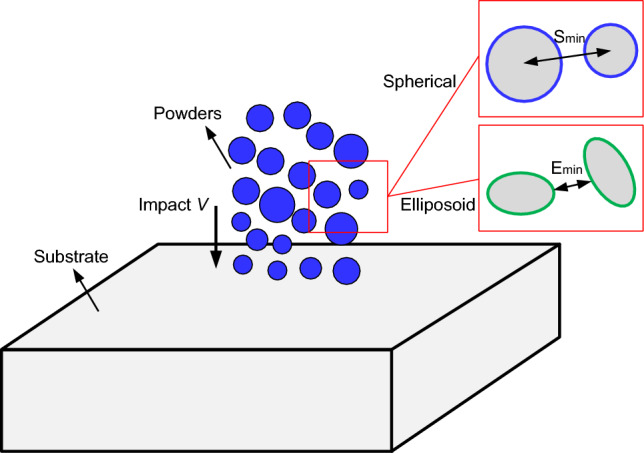
Generation of spherical and ellipsoidal powders using DEM.

Figure 2 shows the schematic of generating spherical and ellipsoidal powders using DEM. The Al-6061 substrate is large enough to ignore the boundary effects. The powders are distributed in a cylindrical area following the rules outlined in Ref.^36^, ensuring non-intersection, the maximal distance to prevent excessive separation between powders, and the minimal distance to prevent powder entrapment. The mathematical expressions of the rules are given as follows:

1. Powders with different sizes (a total number of *N*) are pre-generated based on the experimental volume fraction.
2. *n* powders are already generated in the computational domain, where *R*_*j*_ and *C*_*j*_ represent the radius and center coordinate of a powder *j* (*j* ∈ [1, *n*]), respectively. Then the powder *i* with *R*_*i*_ and *C*_*i*_ is added into the powder group.
3. To satisfy the condition of no intersection, it is given by 1.05 *R*_*i*_ + *R*_*j*, max_ < *S*_*e*, min_(*C*_*i*_, *C*_*j*_), where* R*_*j*, max_ represents the maximum radius of the *n* powders, *S*_*e*_ represents the Cartesian coordinate distance, and *S*_*e*, min_(*C*_*i*_, *C*_*j*_) represents the minimum distance between the powder *i* and its surrounding powder *j*.
4. The maximum distance *D*_max_ between powders should be *D*_max_ > *S*_*e*, max_(*C*_*i*_, *C*_*j*_), where *S*_*e*, max_(*C*_*i*_, *C*_*j*_) denotes the maximum distance between the powder *i* and *j*. *D*_max_ is defined as *D*_max_ = *S*_*e*_(*C*_*i*_, $\sum\nolimits_{j = 1,n} {\frac{{R_{j} C_{j} }}{{\sum\nolimits_{i = 1,n} {R_{i} } }}}$) + *R*_*i*_ + *R*_*j*, max_.
5. The minimum distance *D*_min_ between powders should be *D*_min_ < *S*_*e*, min_ (*C*_*i*_, *C*_*j*_).

It is worth noting that there are different ways of generating DEM powders for the computation with SPH (particle representation). For instance, the open-source code LIGGGHTS or some software can be used for generating multiple spherical powders and obtaining the ellipsoidal powder configurations. For these ways of powder generation, the volume fraction of powders needs to be consistent with the experimentally obtained values. After generating the powders with DEM, the SPH particles are employed to discretize each of them and conduct the CSAM process modeling.

## Validation of the material model

**Figure 3:**
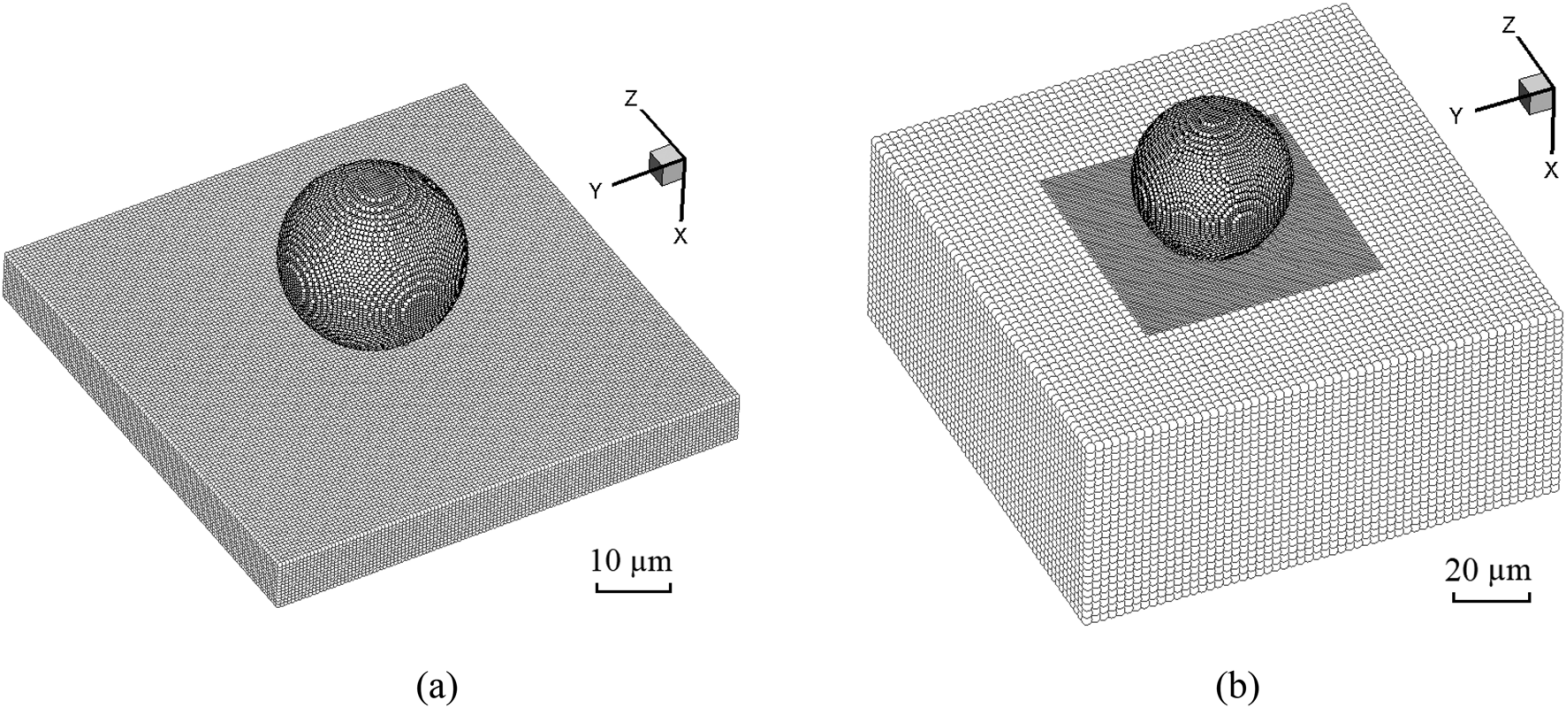
(**a**) Model set-up for the Al-6061 powder impacting on a sapphire surface, and (**b**) multi-resolution model set-up for the Al-6061 powder impacting on an Al-6061 substrate.

**Figure 4:**
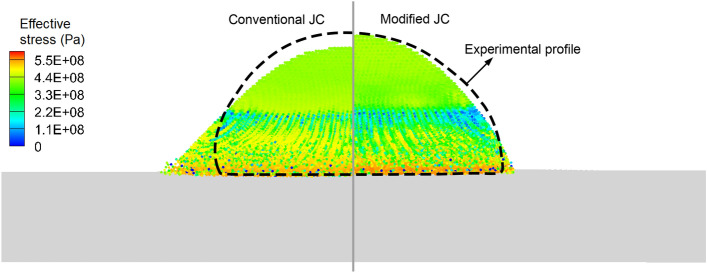
Effective stress of the deformed powder obtained with conventional and modified JC models.

In this section, we first validate the improved SPH method with a modified JC model through simulating a single powder impact case. Figure 3 shows the model set-up for the impact of an Al-6061-T6 powder onto a sapphire substrate (Case I) and an Al-6061-T6 substrate (Case II), respectively. The experimental observations reported by Xie et al.^51^ are adopted for comparisons. In the numerical model, the sapphire and Al-6061-T6 substrates are of sufficient sizes to disregard the effects of boundary constraints. The powder diameter *d*_0_ ranges from 17.0 to 22.6 µm, and the powder velocity *V* varies from 290 to 1130 m/s in these two cases. The speed of sound in air is *c* = 340 m/s, and the dimensionless powder velocity is defined as *V*_*d*_ = *V*/*c*. As shown in Fig. 4, the deformation of the powder in Case I obtained by the conventional JC and the modified JC are compared. It is seen that the modified JC model effectively captures the strain-rate hardening effects while introducing smaller material deformation.

**Figure 5:**
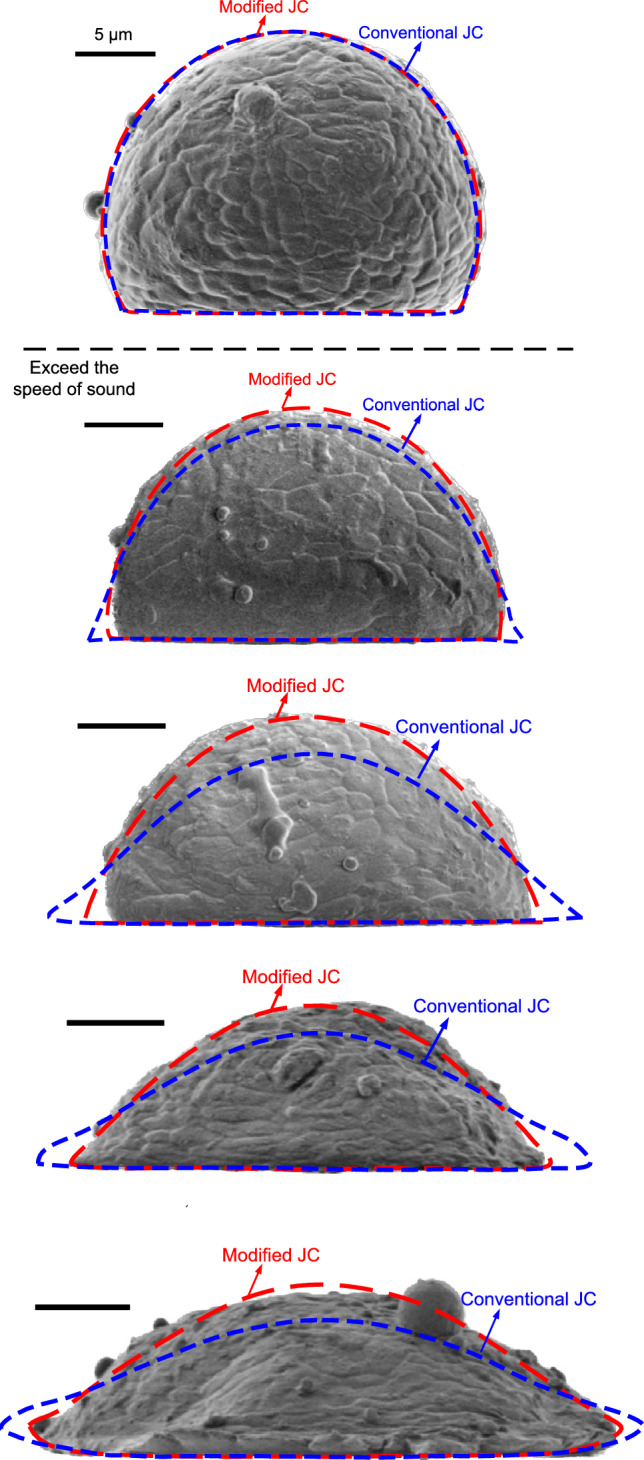
Deformation of an Al-6061 alloy powder after collision to sapphire surface, obtained by experiments^51^ and present simulations. From up to down, *V*_*d*_ = 0.85, 1.24, 1.56, 1.74, and 1.94.

**Figure 6:**
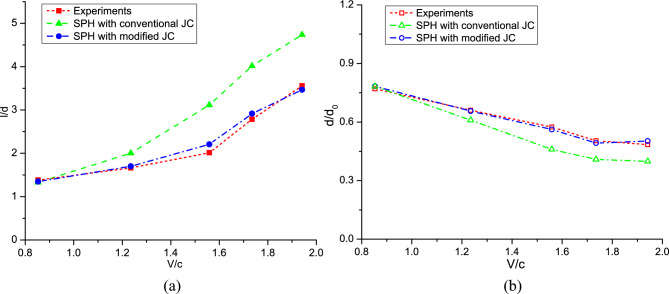
Comparison of the powder deformation parameters between results obtained from experiments^51^, and present SPH simulations with modified and conventional JC models, (**a**) *l*/*d* and (**b**) *d*/*d*_0_.

In Fig. 5, the contours of the deformed powders from experiments and corresponding simulations are comparatively displayed. The sapphire substrate experiences minimal deformation and is thus not displayed in the figures. When the impact velocity is lower than the speed of sound, both the conventional and modified JC models yield results in good agreement with the experimental observations. However, as the impact velocity increases, the conventional JC model fails to accurately predict powder deformation because of its inability in capturing material deformation with a strain rate exceeding 10^3^ s^−1^. In contrast, the modified JC model successfully obtains profiles of powder deformations that closely resemble the experimental data, spanning from subsonic to supersonic regimes. Figure 6 shows a quantitative comparison of the powder deformation parameters obtained from different approaches. *l* and *d* represent the maximum and minimum diameters of the deformed powder, respectively. When the impact velocity exceeds 1.2*c*, the conventional JC model becomes unreliable, particularly for *V*_*d*_ > 1.6. In summary, these comparisons highlight the necessity of employing an appropriate material model to predict strain-rate hardening effects in CSAM modeling.

**Figure 7:**
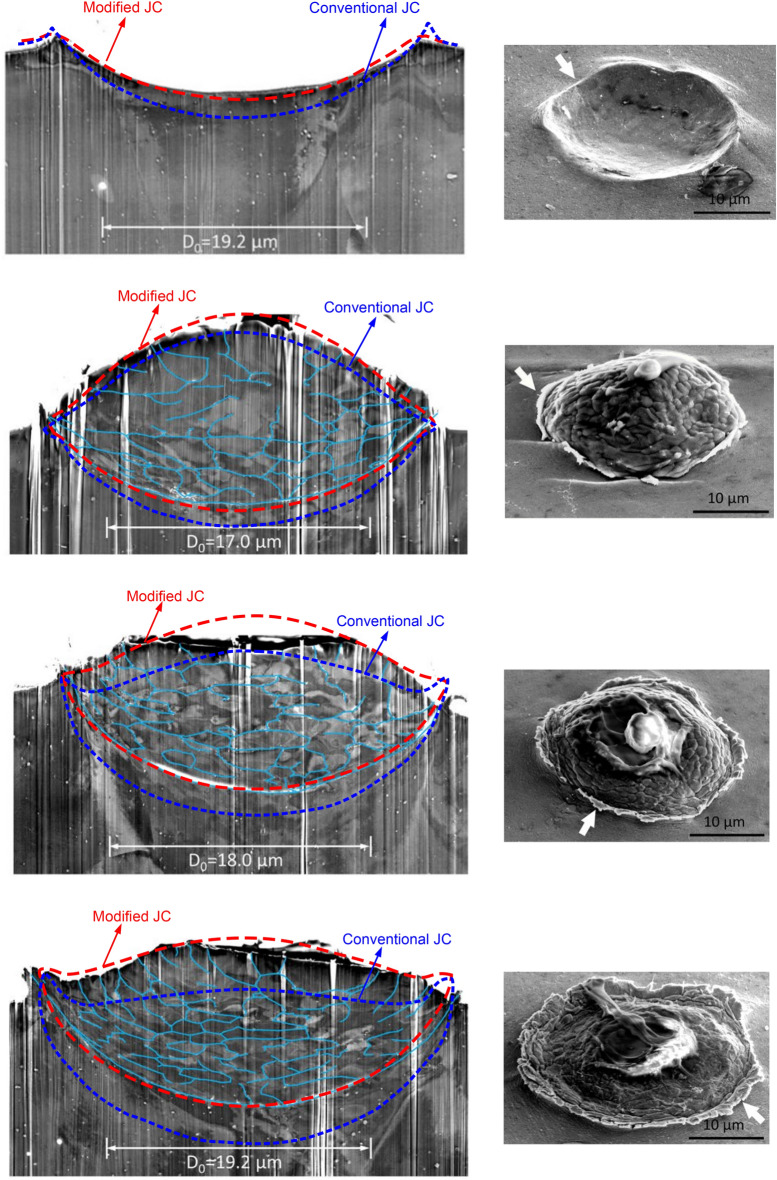
Deformation of the Al-6061 alloy powder and substrate obtained by experiments^51^ and present simulations. From up to down, *V*_*d*_ = 2.35, 2.65, 2.94, and 3.32.

**Figure 8:**
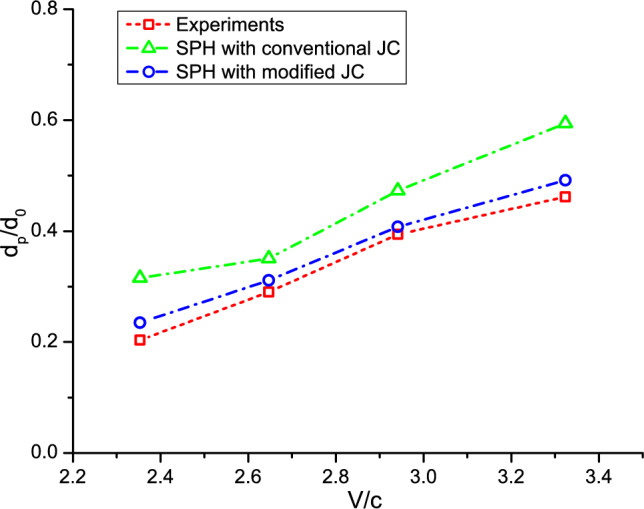
Comparison of the crater depth between results obtained from experiments^51^, and present SPH simulations with modified and conventional JC models.

Furthermore, the impact of an Al powder on an Al substrate is simulated, allowing observation of the resulting deformation in the substrate. Figure 7 shows the crater formed on the substrate obtained from different sources. The conventional JC model predicts a deeper intrusion due to significant material deformations in the numerical simulations. On the contrary, a satisfactory crater shape is obtained by the modified JC model. Upon increasing the impact velocity, we observe enhanced powder deformations with evident pile up. It shows the advantage of the present particle modeling over mesh-based methods, which struggle to replicate such phenomena. It is seen that the modified JC results agree well with the experimental ones across a wide range of impact velocities. Moreover, a quantitative comparison regarding the coating depth *d*_*p*_ is presented in Fig. 8. The numerical results obtained by the conventional JC exhibit more deviations from the experimental data as the impact velocity increases. It is important to mention that the conventional JC model offers less accurate results at a lower impact velocity of 2.35*c*, which is attributed to the smaller value of the reference coating depth. The validation tests in this section confirm that the present modified numerical method reliably predicts deformations of the powder and substrate at high strain rates in CSAM.

## CSAM with different powder sizes, morphologies, and distributions

### CSAM of spherical powders with different sizes

**Figure 9:**
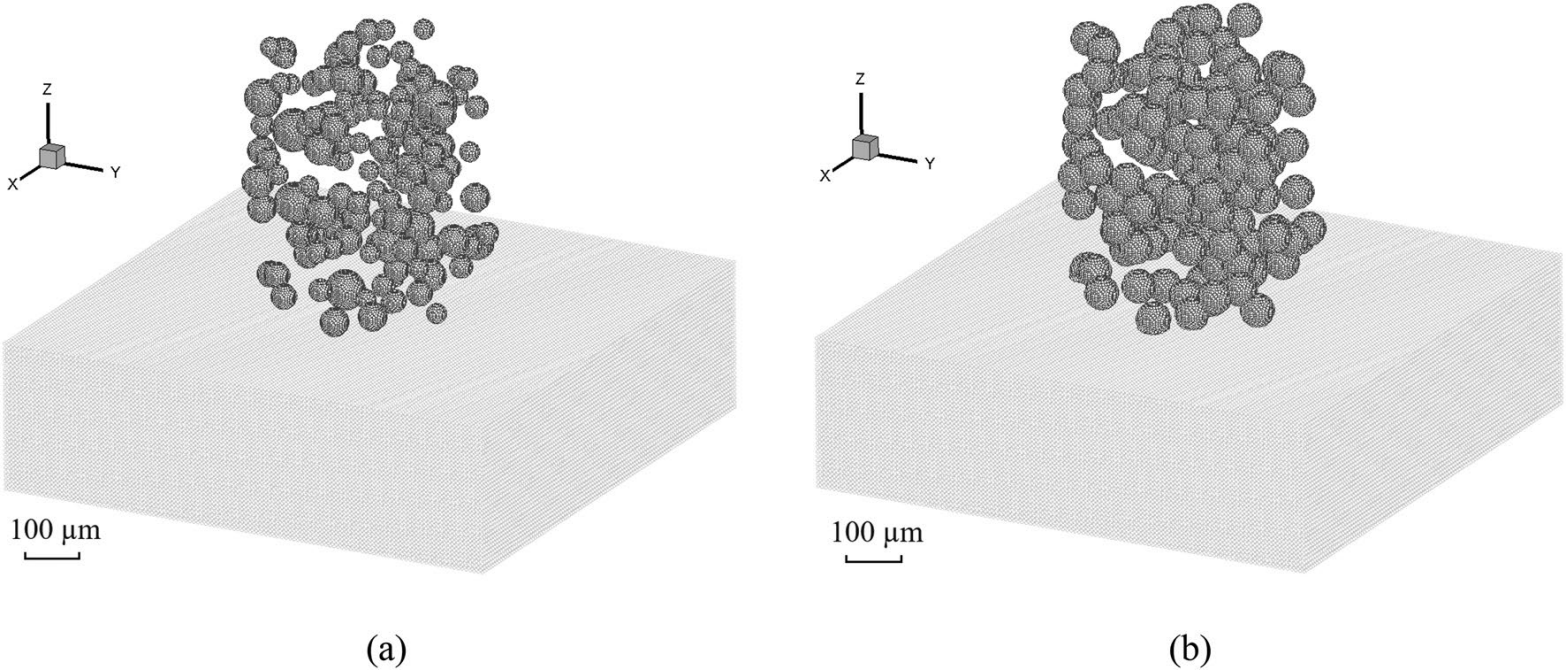
Model set-up for CSAM with (**a**) different powder sizes obtained from experiments^52^ and (**b**) a uniform powder size. The volume fractions of powders in these two cases are the same.

In this section, the CSAM of spherical powders with different sizes are first modeled and evaluated. Figure 9a shows the model set-up of the powders and substrate based on the experimental volume fraction^52^. The positions and sizes of the powders are obtained using the aforementioned DEM model, and each powder is discretized by a number of SPH particles. A model of powders with a uniform size, *d*_0_ = 36.3 µm in Fig. 9b, is also considered for the purpose of comparison, where the overall volume and number of powders are the same as those in the experiments. In CSAM operations, powders can be accelerated to speeds ranging from 300 to 1200 m/s through the application of gas pressure^54^. To attain a successful coating in this work, the impact velocities of the powders are adopted from *V* = 500–1100 m/s, with an interval of 100 m/s.

**Figure 10:**
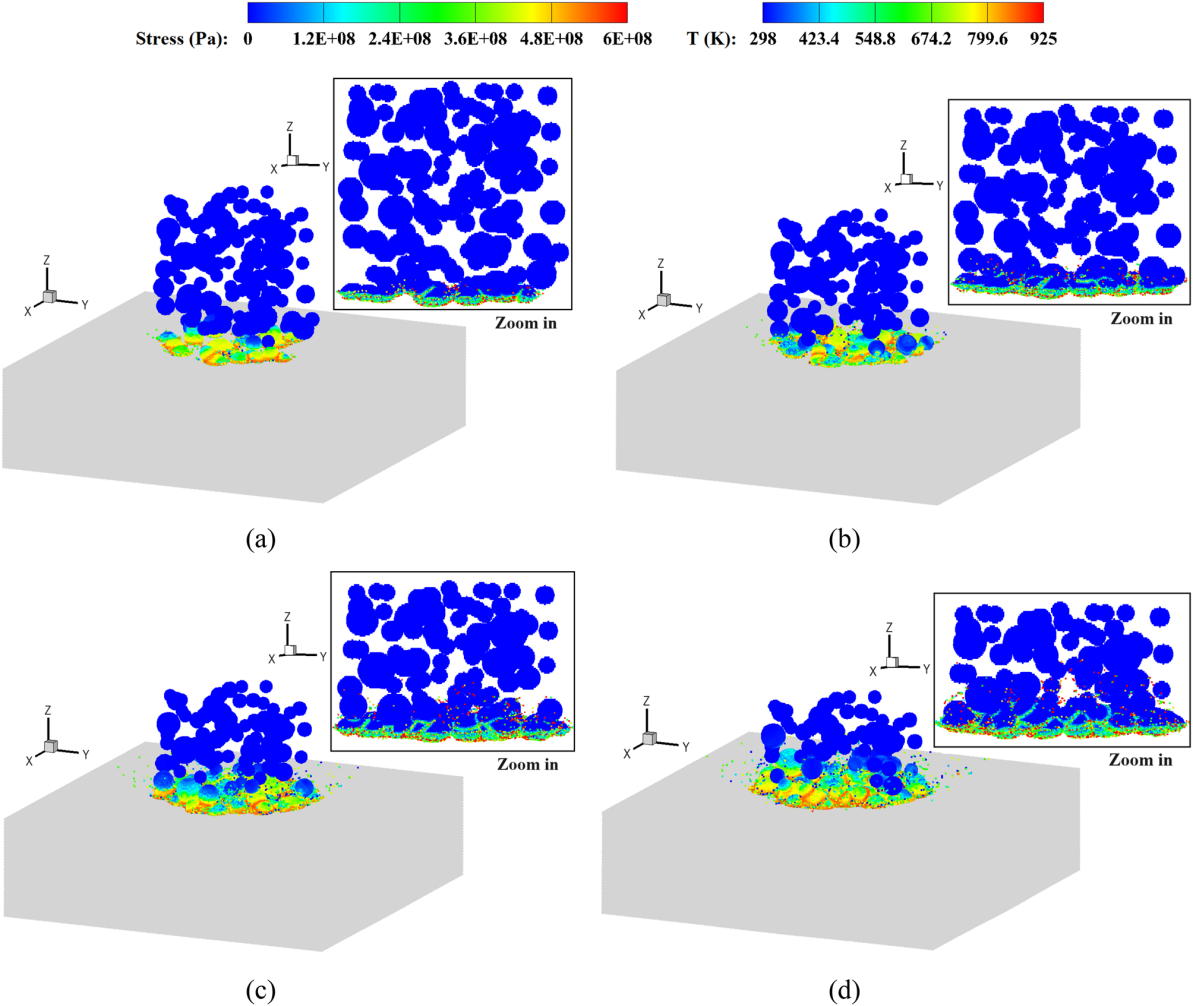
Stress field and temperature field (zoom in view) in deposited powders with different sizes, *V*_*d*_ = 2.65. *t* = 50 (**a**), 100 (**b**), 150 (**c**) and 200 (**d**) ns.

**Figure 11:**
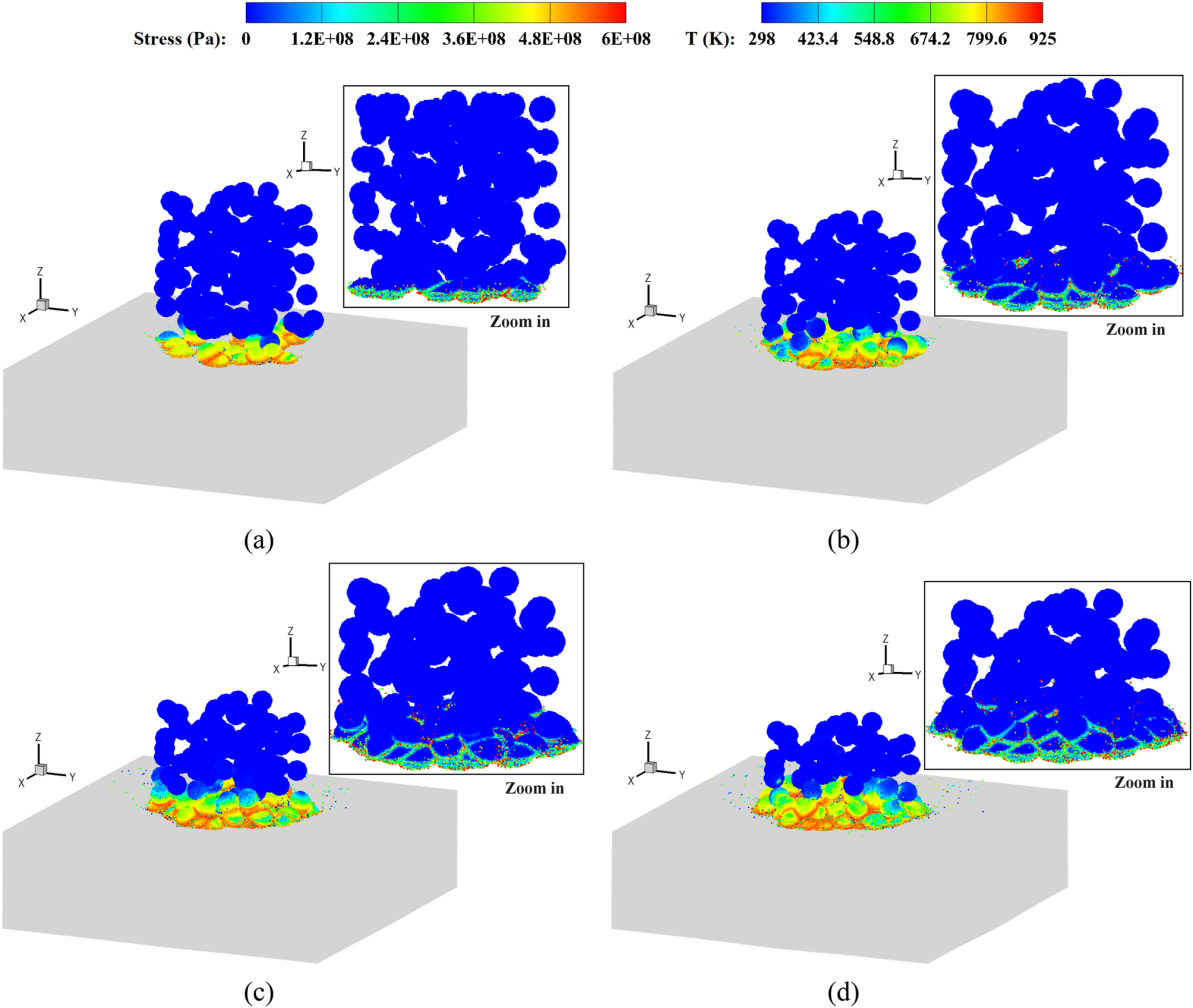
Stress field and temperature field (zoom in view) in deposited powders with a uniform size, *V*_*d*_ = 2.65. *t* = 50 (**a**), 100 (**b**), 150 (**c**) and 200 (**d**) ns.

**Figure 12:**
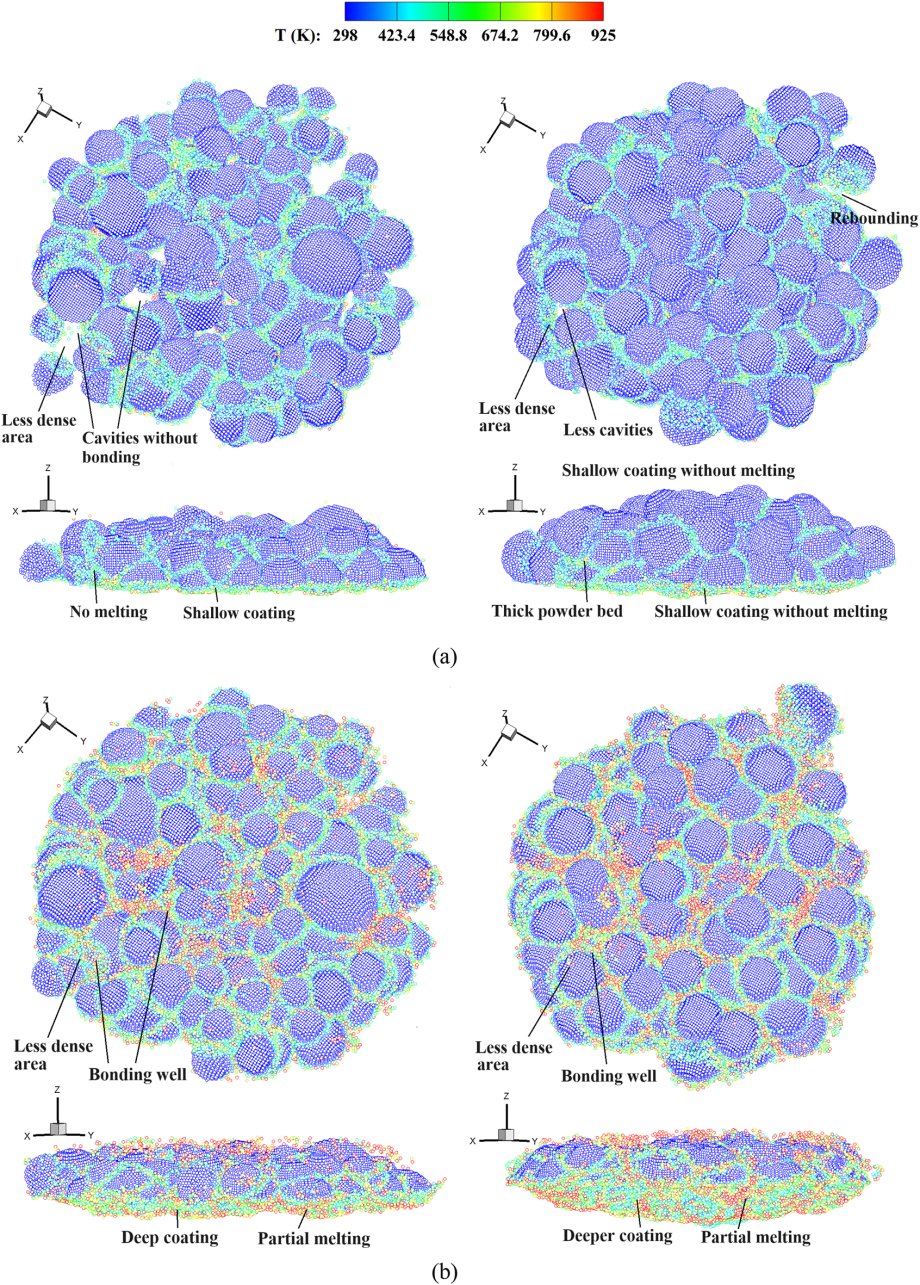
Top and side views of the bonding powders obtained with experimental powder sizes (left) and a uniform powder size (right), (**a**) *V*_*d*_ = 1.47 and (**b**) *V*_*d*_ = 2.65.

Figures 10 and 11 show the stress and temperature fields in the powders during their deposition processes. It is observed that the powders collide with the base plate and get bonded together, where the material melting occurs predominantly in the vicinity of the interface regions. Plastic deformation and jetting formation are well reproduced by the present simulations, which are difficult to capture using mesh-based methods. Upon zooming in, less dense regions can be observed in the powders corresponding to the experimental volume fraction. In contrast, powders with a uniform size exhibit a more densely packed arrangement. Figure 12 provides top and side views of the bonding powders obtained with two typical impact velocities. At a relatively low impact velocity of 1.47*c*, a shallow coating is formed for both types of powder distributions. When the impact velocity is increased to 2.65*c*, a noticeably deeper coating is achieved accompanied by partial melting of the bonding interfaces. It should be noticed that the powders in less dense areas may not effectively be coated together or adhered to the substrate, resulting in cavities between the powders. Such a large porosity is usually undesirable to produce dense components. With an increase in impact velocity, the interfacial porosity becomes smaller as the powders undergo large deformations and begin to grind against each other. In the case with a uniform powder size, a small number of cavities can be observed even at a low impact velocity. This is because small deformations in the super large powder may result in cavities between powders of different sizes. Besides, extremely less dense areas can somehow be avoided in the uniform powder case, and the powders distribute relatively close to each other. From the comparisons, we note that the impact velocity for powders in a practical experimental set-up should be sufficiently high, not only for achieving effective coating but also for preventing interfacial cavities. Moreover, when a uniform powder size is adopted, the interfacial porosity decreases even at a lower impact velocity, thereby enabling the production of a thicker powder bonding bed. The formation of a bonding powder layer, as well as the presence of porosity, demonstrates a strong dependency on the sizes and distributions of the powders. However, evaluating this dependency becomes challenging when using powders of a uniform size arranged in a staggered pattern, which has been performed in most research work.

**Figure 13:**
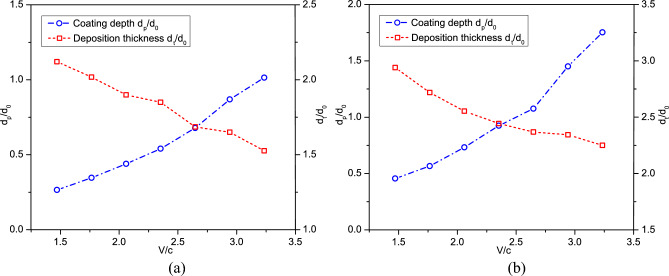
Variation of deposition thickness and coating depth versus impact velocity obtained with (**a**) experimental powder sizes and (**b**) a uniform powder size.

**Figure 14:**
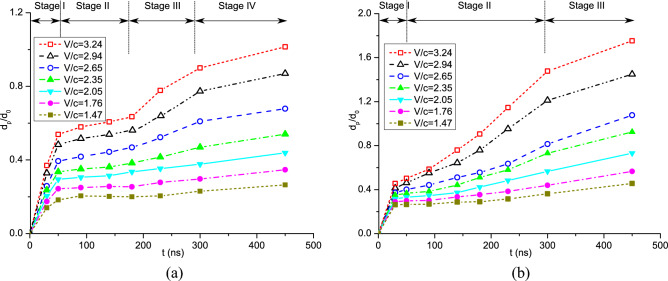
Time histories of the coating depth at different impact velocities obtained with (**a**) experimental powder sizes and (**b**) a uniform powder size.

Furthermore, the variations of deposition thickness and coating depth versus impact velocity are shown in Fig. 13. The coating or intrusion depth increases dramatically when the impact velocity is larger than 2.5. This is due to the large deformations of powders at higher impact velocities. On the other hand, the deposition thickness decreases linearly as the impact velocity increases. CSAM with a uniform powder size produces a deeper coating on the substrate and a thicker powder layer than that with different powder sizes. Next, we compare the time histories of coating depth for these two cases in Fig. 14. For CSAM utilizing experimental powder sizes, a four-stage variation of coating depth can be observed. Conversely, when a uniform powder size is adopted, the variation of coating depth exhibits three stages, where the second stage of the latter case cannot be observed.

**Figure 15:**
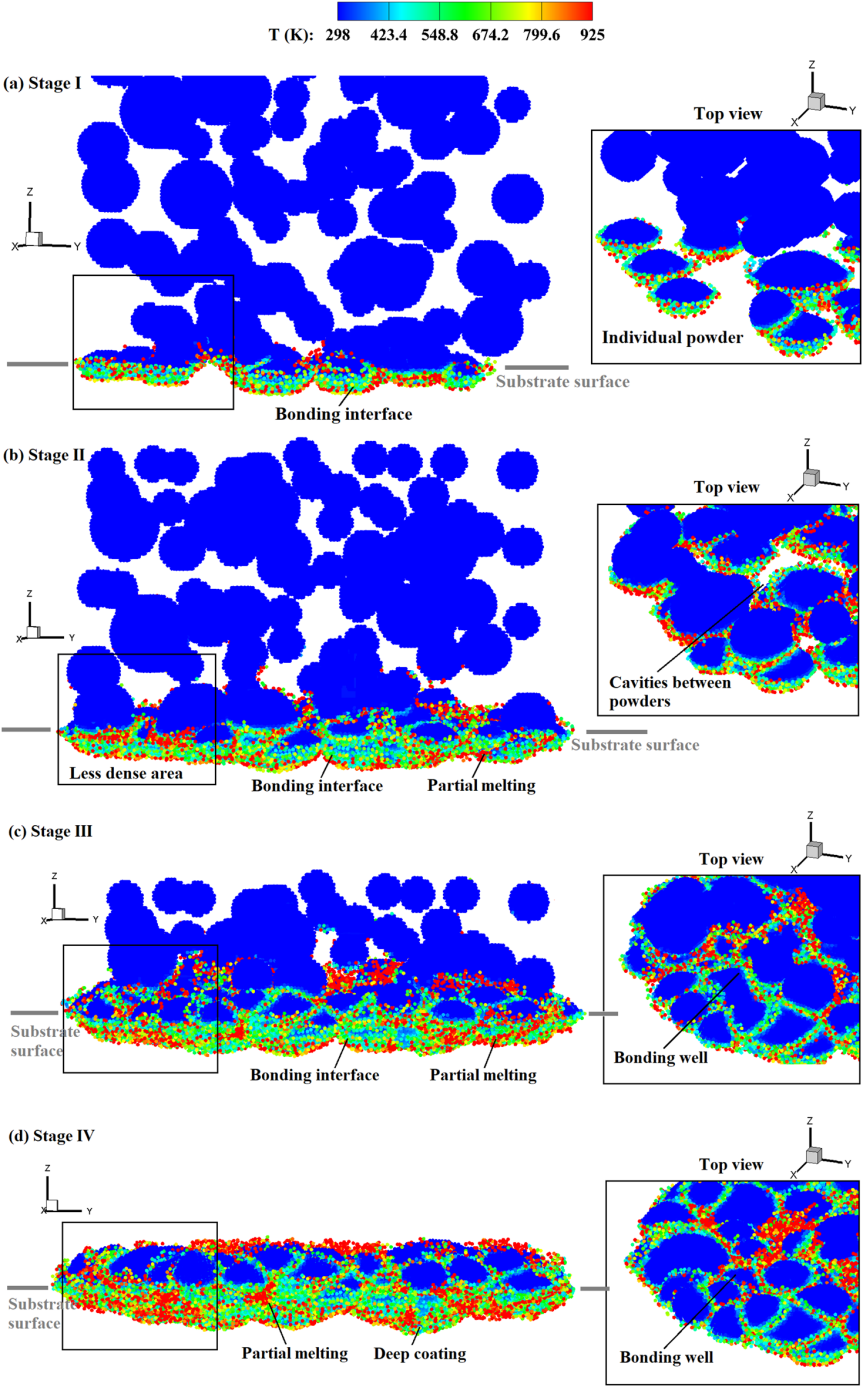
Temperature distribution in the powders during the CS process with experimental powder sizes, *V*_*d*_ = 2.94, *t* = 45, 135, 225, and 300 ns (corresponding to four stages).

Figure 15 shows the temperature distribution in the powders during the CS process comprising four stages. In the first stage, the coating depth increases rapidly with the impacting of powders around the first layer, and the increase rate is dependent on the impact velocity. As depicted in Fig. 15a, the powders individually impact onto the substrate during this stage. In the second stage (50–200 ns), more powders are sprayed onto the substrate, filling the gaps or cavities between them. During this stage, the first layer of bonding powders is gradually formed, and the coating depth increases slowly, just as shown in Fig. 15b. After this, only a very limited number of cavities between bonding powders can be observed in the less dense area. In stage III, as the coating progresses, the powders continuously impact onto the previous layer of powders. The coating depth shows an evident growth trend again, whereas the increasing rate is lower than that of the first stage. As shown in Fig. 15c, partially melted interfaces without cavities are produced in this stage. When the majority of powders impact onto the substrate, i.e., stage IV, the coating depth increases slowly and finally reaches a maximal value, as seen in Fig. 15d. For CSAM with a uniform powder size, stage II is very short or may even not be recognized. This is attributed to the scarcity of cavities between different powders, which allows for rapid formation of the first layer of bonding powders. Therefore, variations in the coating depth directly goes into the subsequent phase of rapid increase. Through both qualitative and quantitative comparisons, the formation of bonded particle layers can be well elucidated.

### CSAM with different powder morphologies

**Figure 16:**
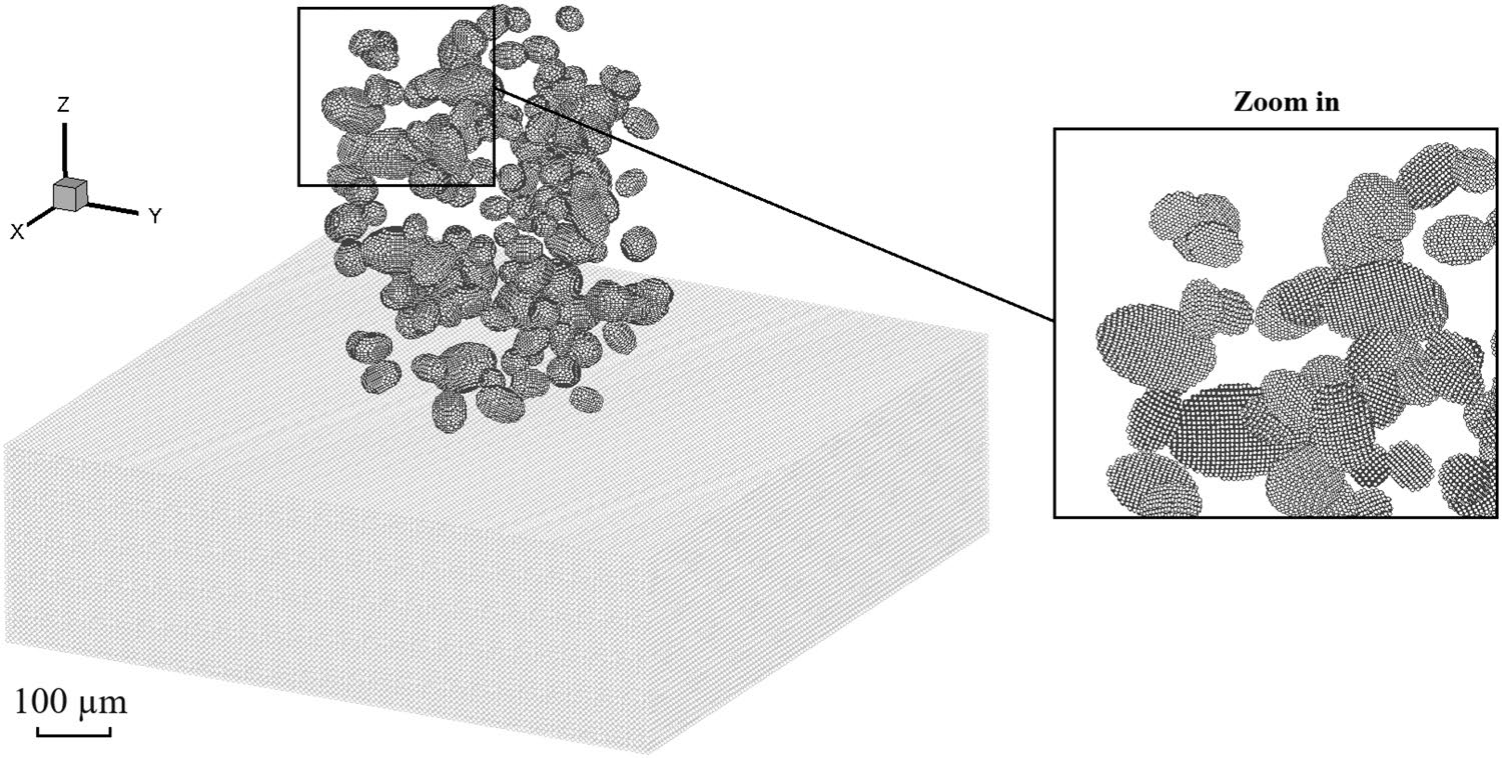
Model set-up for CSAM of ellipsoidal powders with different sizes, *a*/*b* = 1.5.

The powder morphology is usually determined by the powder production technique, e.g., the gas atomization technique often produces spherical and ellipsoidal powders^17^. Moreover, when temperature of the carrier gas in CS is increased, the morphology of the powders becomes progressively more irregular. This phenomenon was substantiated by Wang et al.^55^, who demonstrated that at a notably elevated gas temperature of 300 ºC, distinct ellipsoidal powders could be observed. In view of this, we further evaluate the influences of powder morphology on the bonding of Al-6061 powders with a substrate. Figure 16 presents the distributions of ellipsoidal powders with different sizes and orientations. The powder volume fraction is the same as in the experimental case mentioned above, except that ellipsoidal powders are adopted. The lengths of the three axes of an ellipsoid are defined as *a*, *b*, and *c*, where* b* equals *c* to avoid the occurrence of specially flattened ellipsoids. The orientation of the principal axis of an ellipsoidal powder is randomized, and the ratio of *a* to *b* varies from 1.0 to 1.5 to adjust the morphology of an ellipsoid. The powders are placed in a cylindrical area with appropriate distances without intersection, i.e., following the same rules as for generating the spherical powders^36^.

**Figure 17:**
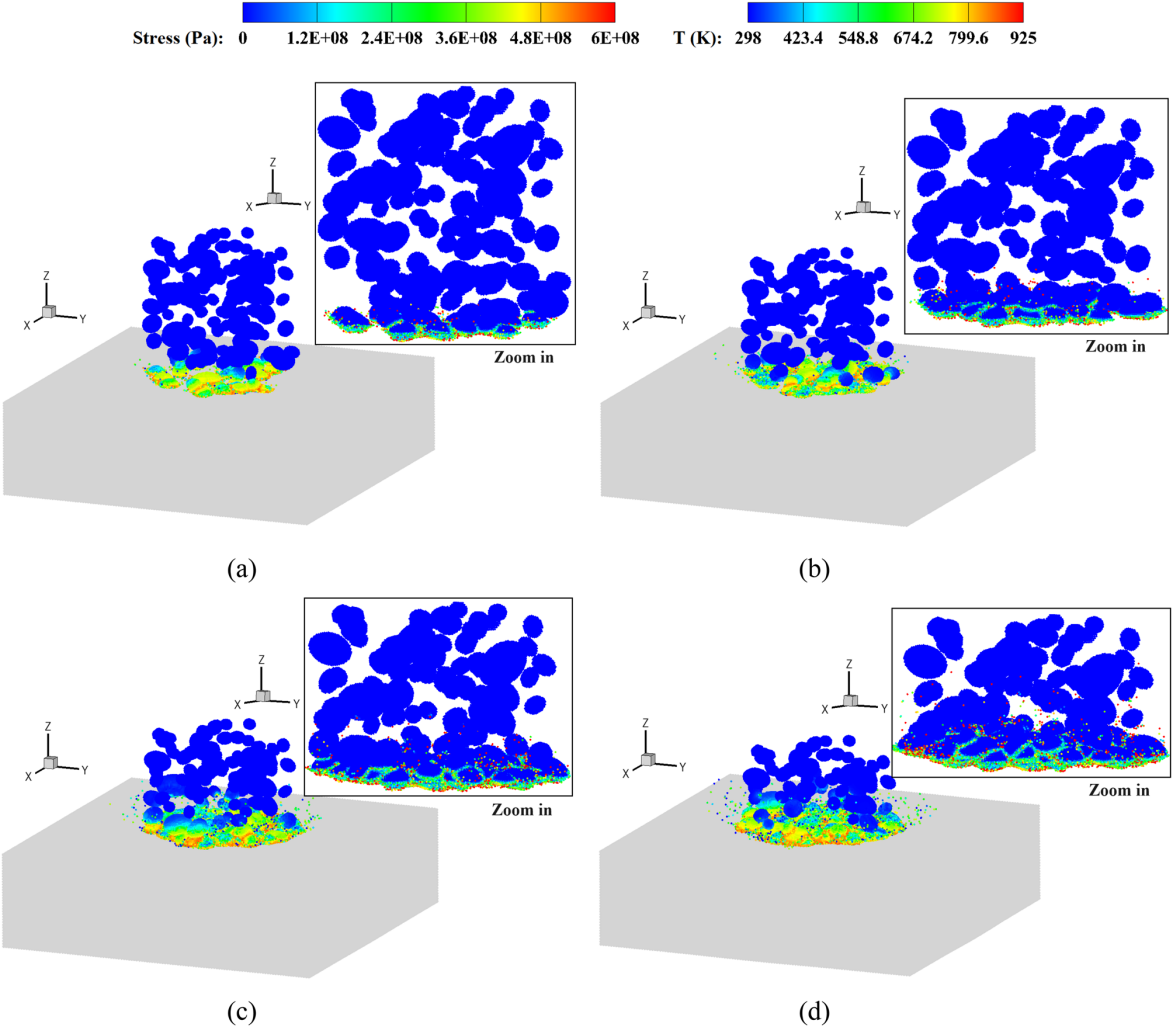
Stress field and temperature field (zoom in view) in deposited ellipsoidal powders with different sizes, *V*_*d*_ = 2.65. *t* = 50 (**a**), 100 (**b**), 150 (**c**) and 200 (**d**) ns.

**Figure 18:**
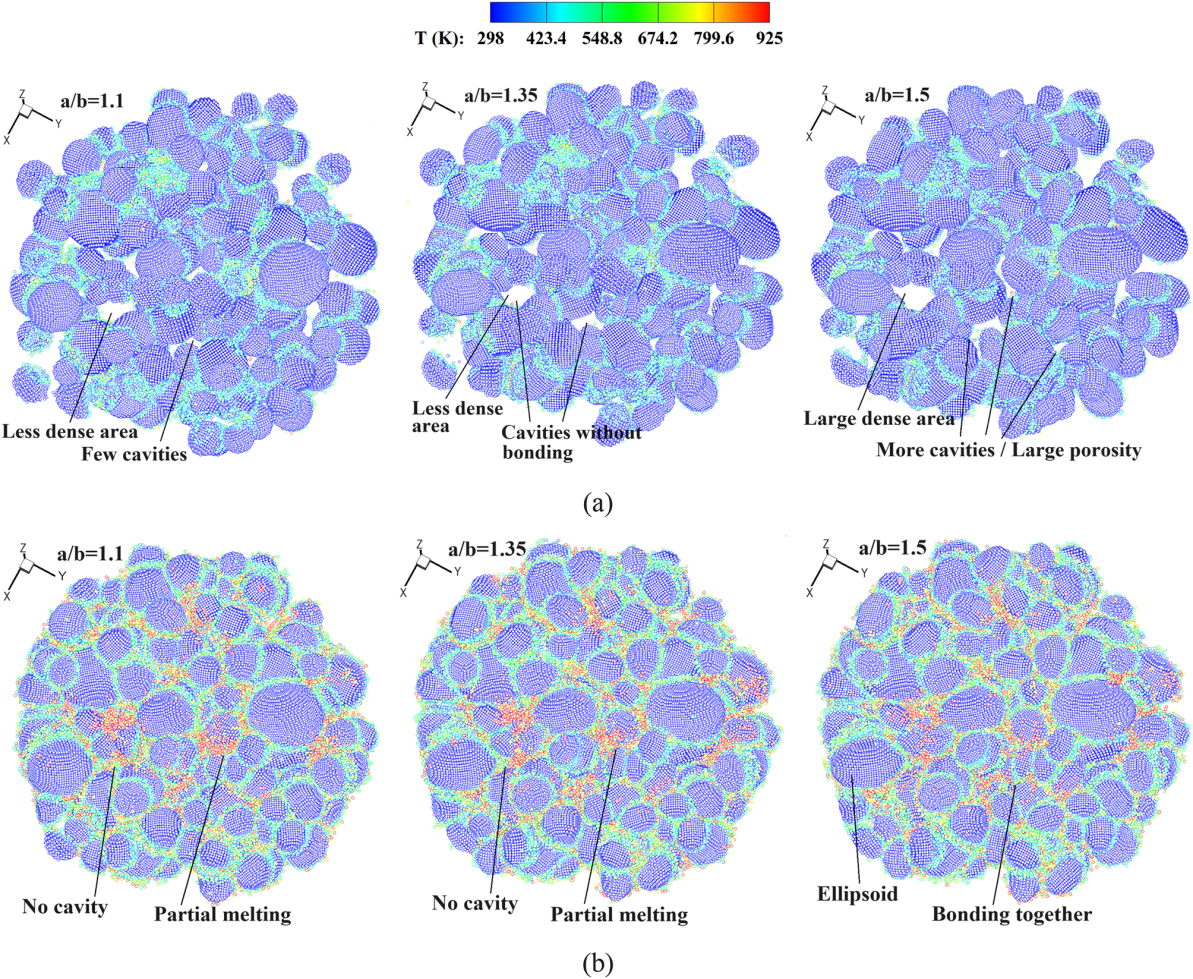
Top views of the bonding ellipsoidal powders with different ratios of length to diameter, (**a**) *V*_*d*_ = 1.47 and (**b**) *V*_*d*_ = 2.65.

Figure 17 shows stress and temperature fields for the deposited ellipsoidal powders of varying sizes. It is observed that at high impact velocities, bonding between the powders and the substrate is achieved with partially melted interfaces. In this case, the coating becomes much more intricate as the ellipsoidal powders are distributed with different sizes and orientations, resulting in a larger porosity within the bonding powder bed. As shown in Fig. 18, an increased length-to-diameter ratio (*a*/*b*) leads to a greater presence of cavities between bonding powders. Oppositely, a smaller porosity is evident in the case with spherical powders, as depicted in Fig. 12a. When the impact velocity is increased to *V*_*d*_ = 2.65, the ellipsoidal powders effectively bond together, and the length-to-diameter ratio exhibits minimal influence on the porosity of the powder bed. According to the findings of Weiller and Delloro^36^, the size and spatial configuration of the powders exert a substantial influence on the resulting porosity. The introduction of ellipsoidal powders significantly augments the complexity of their spatial arrangements and gives rise to pronounced regions of a reduced density. Additionally, the distinct orientations of the ellipsoid axes facilitate the formation of cavities at the contact regions between powders, particularly at lower impact velocities.

**Figure 19:**
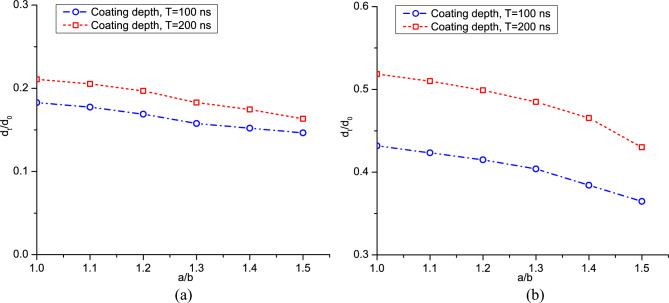
Variation of the coating depth versus length-diameter ratio of the powders, (**a**) *V*_*d*_ = 1.47 and (**b**) *V*_*d*_ = 2.65.

In Fig. 19, we present a comparison of variations in the coating depth across different length-diameter ratios. The coating depth decreases with an increase in *a*/*b*, which is more pronounced in cases with larger impact velocities. Notably, when the length-diameter ratio surpasses 1.1, the coating depth experiences a substantial drop. For cases with impact velocities *V*_*d*_ = 1.47 and *V*_*d*_ = 2.65, the coating depth for ellipsoidal powders could be smaller than 0.2 *d*_0_ and 0.45 *d*_0_, respectively, highlighting the significant influence of the ellipsoid degree. In the spherical powder impacting case, the kinetic energy of a powder is consumed by its plastic deformation and is transmitted to the internal energy in a straightforward way. However, an ellipsoidal powder may rotate and rebound upon impacting on a substrate, which may lead to lower coating depths. Consequently, to achieve an optimal coating between the powders and the substrate, it is recommended to maintain the ellipsoid degree within a range, preferably smaller than 1.1.

**Figure 20:**
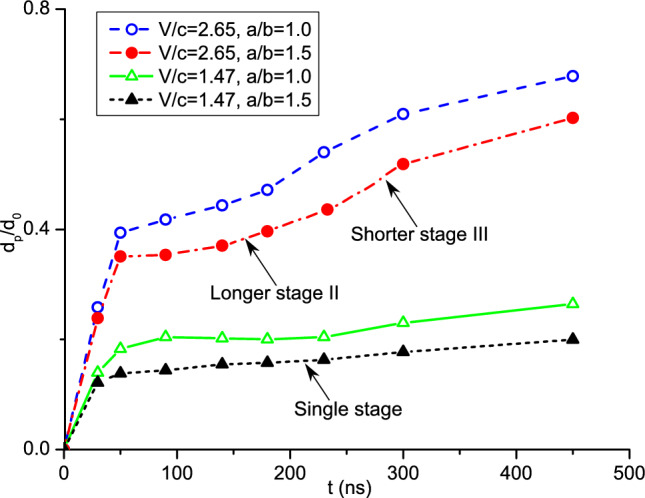
Comparison of the coating depth obtained by CS with spherical and ellipsoidal powders.

**Figure 21:**
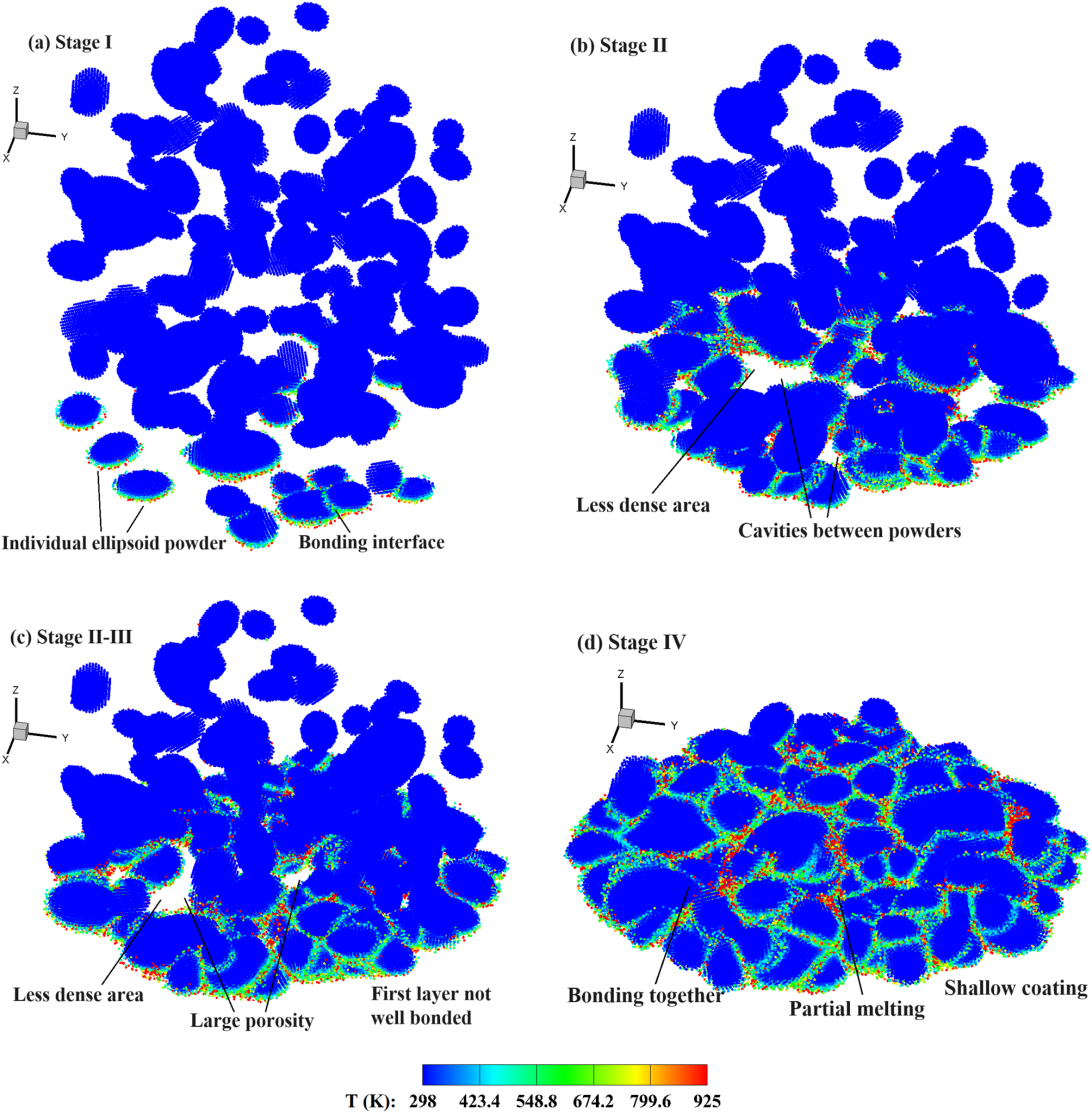
Temperature distribution in the ellipsoidal powders during the CS process, *a*/*b* = 1.5, *V*_*d*_ = 2.65, and *t* = 40, 130, 180, and 300 ns (corresponding to different stages).

Furthermore, we analyze the effects of the length-to-diameter ratio on the formation of the bonded powder layer. Figure 20 presents the temporal evolution of the coating depth in CS with spherical and ellipsoidal powders. In the case involving spherical powders, we have identified a four-stage variation of the coating depth, which is also applicable to the ellipsoidal case. During the second stage, the coating depth increases slowly as it requires a certain amount of time for the powders to bond together and form the initial layer of the powder bed. In the case of ellipsoidal powders, stage II is prolonged, while stage III is shortened. This phenomenon is elucidated by Fig. 21, which illustrates the coating process of the ellipsoidal powders. At *t* = 180 ns, visible cavities are still present between powders, not only in the less dense areas. The large porosity at this stage arises due to the configuration of the ellipsoidal powder. Consequently, a longer duration is needed to establish a dense powder layer, resulting in an elongated stage II. When the impact velocity is lower, specifically *V*_*d*_ = 1.47, the coating depth increases slowly in the ellipsoidal powder case, without exhibiting a multi-stage increment pattern. This is attributable to the fact that the impact velocity is not large enough for the ellipsoidal powders to fill the cavities between powders, thereby leading to a persistent high powder porosity throughout the coating process. Such a variation of coating depth at lower impact velocities is in good consistence with the observed porosity of the powders illustrated in Fig. 18.

### Complete CSAM process with experimental powder sizes and distributions

**Figure 22:**
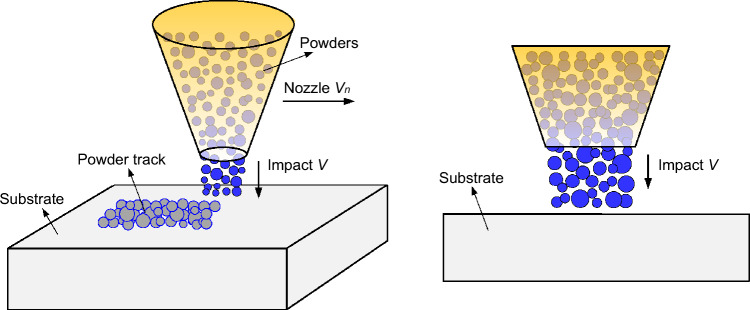
Model illustration for the multi-layer multi-track CS with experimentally obtained powder sizes and distributions.

Finally, the present model is applied to reproduce a complete CSAM process with multi-layer multi-track coatings, aiming to show the capability of the parallel meshless computational scheme and enhance our understanding of bonding mechanisms at different tracks. Figure 22 provides different views of the numerical model, wherein powders with experimental sizes and volumes are sprayed from the nozzle and impact onto the substrate. Both the powders and substrate are composed of Al-6061. Again, the substrate is of considerable size to mitigate boundary effects. The spatial distributions of the powders follow the same rules outlined in the previous section. For the single-track coating, the nozzle traverses from the left to the right side of the substrate, and subsequently returns for the second-track coating. The scanning length is set as 1.5 mm. To ensure the formation of a densely packed powder bed, no intervals are introduced between consecutive scanning paths. To reproduce the multi-track CS process, approximately 10 million SPH particles are employed to discretize the powders (numbering in the tens of thousands) and the substrate. This large-scale simulation of the CSAM process is believed to be the highest resolution of its kind to date, elevating the maximum number of discretization elements in the current state of the art.

As reported by Ozdemir et al.^56^, the deposition rate in practical CSAM experiments may reach 80 g/min. In the present numerical modeling, we adopted a higher volumetric build rate to save computational time, which is a common practice in most numerical simulations of CSAM. This is a limitation of the present numerical model. However, our simulations operate on a scale of mm, which means that the powder particles are not in a very dense distribution. While a rate of over 10 powders per nanosecond is deposited on the substrate, they are dispersed over a large area. Hence, when the subsequent powders impact the preceding ones, most of the former powders have already attained their maximum deformations. With this aspect in view, the effect of the thermal history of the deposit could be significantly diminished. Nevertheless, investigating the thermal history of the deposit remains a compelling research direction. It is noted that our current simulations only used tens of CPU cores. When hundreds of CPU cores are used, the practical volumetric build rate could be considered. The developed numerical solver exhibits great potential in addressing this challenge, making it a promising area for future research.

We first show a testing example to outline the computational time required by our current meshless solver in Table 2. Notably, simulations for 1400 powders, discretized by millions of SPH particles, could be completed within a single day using 8 cores. For simulations involving tens of thousands of powders, more cores can be utilized to ensure that the simulation is conducted within several days. Besides, the simulation time does not increase linearly with an increase in the powder number, as the substrate remains fixed and is discretized by one million SPH particles. The present meshless scheme proves highly efficient for considering relatively large-scale (mm) simulations, as opposed to those at the powder scale (µm).

**Table 2:** The computational effort of the particle solver for high-fidelity CSAM simulations.

Physical time *t* (ns)	8 cores					
	No. of powder particles	No. of SPH particles	Simulation time (min)	No. of powder particles	No. of SPH particles	Simulation time (min)
100	700	884,886	231	1400	1,661,668	325
200	700	884,886	469	1400	1,661,668	657
200	700	884,886	905	1400	1,661,668	1236

**Figure 23:**
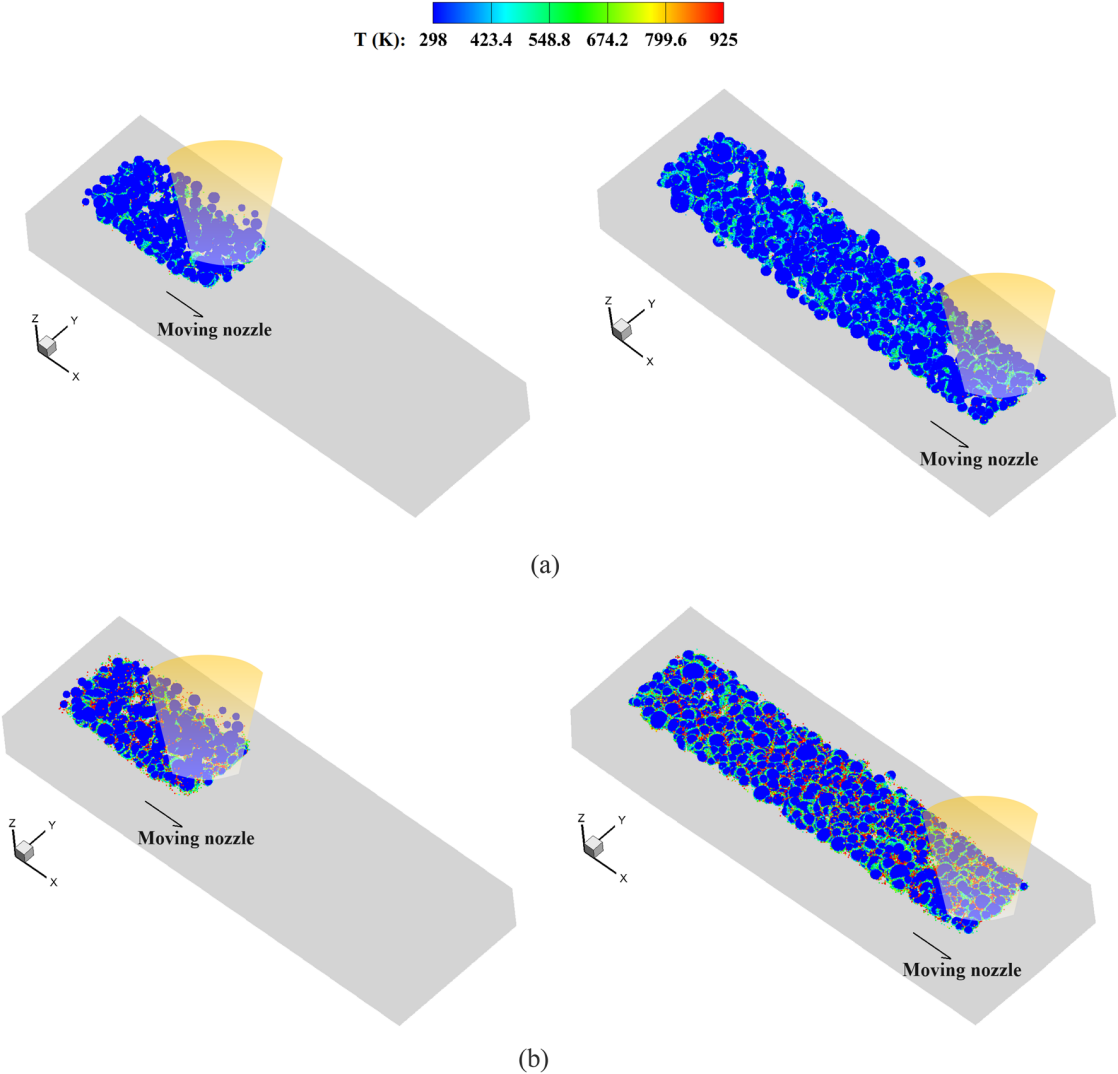
Temperature field in deposited powders during the single-track multi-layer CS process, (**a**) *V*_*d*_ = 1.47, and (**b**) *V*_*d*_ = 2.65.

**Figure 24:**
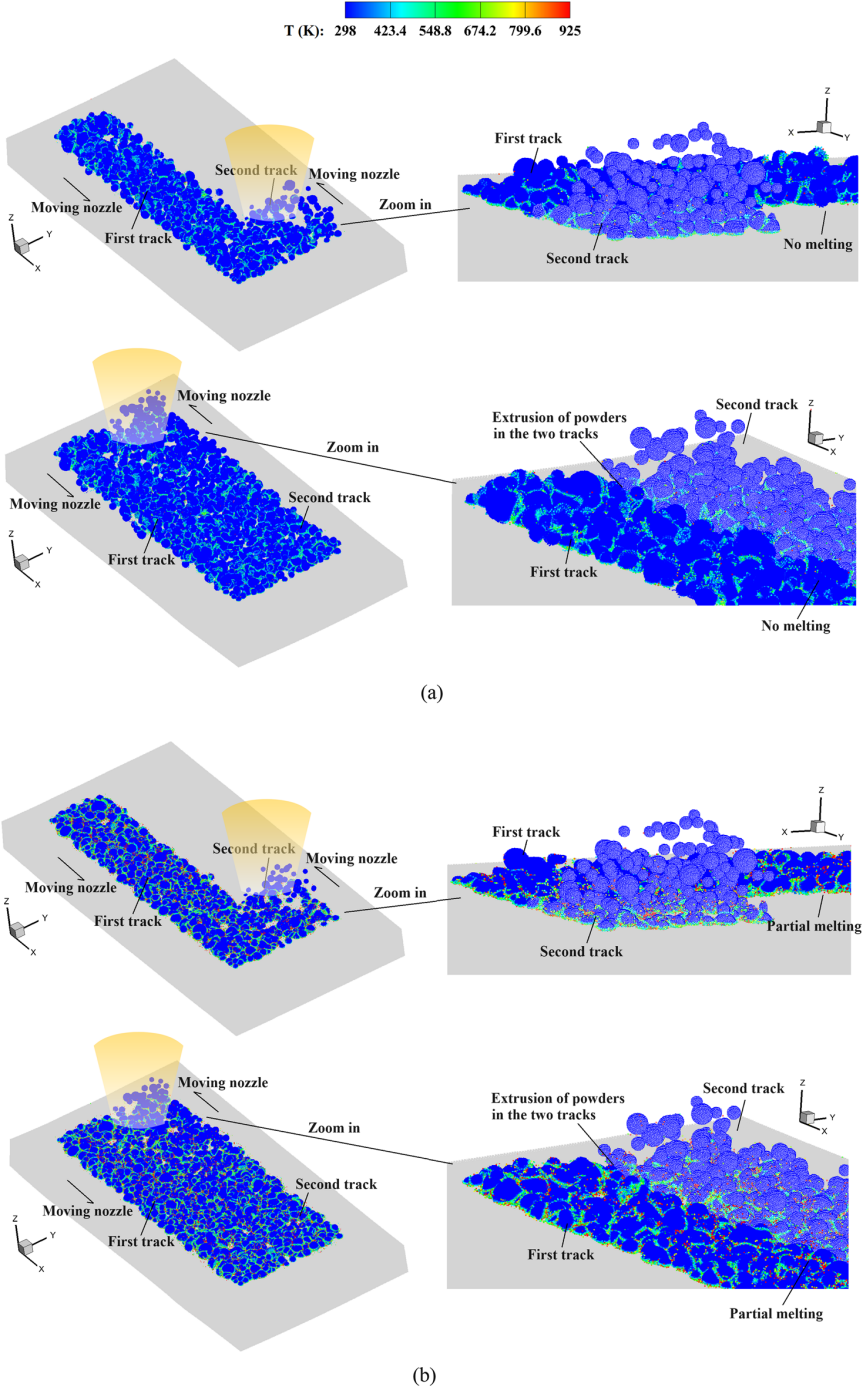
Temperature field in the deposited powders during the multi-track multi-layer CS process, (**a**) *V*_*d*_ = 1.47, and (**b**) *V*_*d*_ = 2.65.

Figure 23 shows the temperature field in the deposited powders during the single-track multi-layer CS process. The coating for a single-track CS is reproduced by the present method, where a larger impact velocity may lead to partial melting of the bonding interfaces. Then, we provide the details of powder distribution during the multi-track multi-layer CS process in Fig. 24. Observations reveal that, as the nozzle moves, powders of varying sizes are coated to one another, forming the initial powder track. In the second track, the powders sprayed from the nozzle impact onto the substrate adjacent to the first track. This process entails the extrusion of powders between different tracks, which facilitates a secure bond between them. The spacing between two scanning paths has great influences on the bonding of two powder tracks. If the spacing is too large, the powders between different tracks may not be coated together. Conversely, when the spacing is too small or overlapping, the bonding of powders in the first track near the interfaces may be destructed, thus resulting in highly irregular powder track surfaces. The extrusion effects of distinct tracks are further manifested in their different coating depths, which will be elaborated on subsequently.

**Figure 25:**
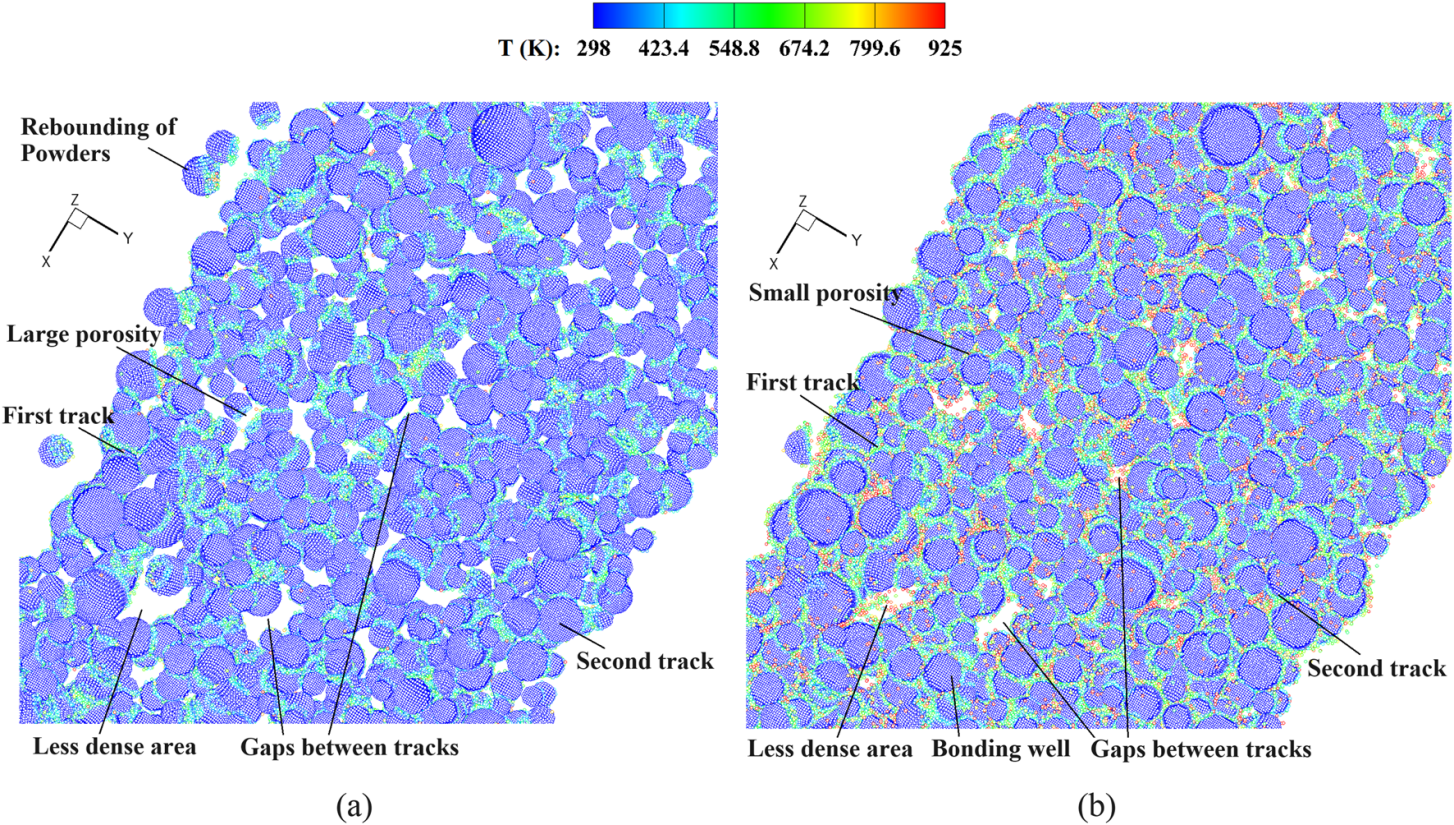
Top views of the deposited powders in the multi-track multi-layer CS. (**a**) *V*_*d*_ = 1.47 and (**b**) *V*_*d*_ = 2.65.

In Fig. 25, the deposited powders in the multi-track multi-layer CS with varying powder impact velocities are shown. For the CS with an impact velocity of *V*_*d*_ = 1.47, the cavities between powders are distributed throughout the entire powder track. In contrast, a higher impact velocity leads to reduced porosity within the powder track, indicating improved quality of the produced components. The inter-powder cavities can only be observed in the less dense areas due to the initial powder distribution from the nozzle outlet. At such a large impact velocity of *V*_*d*_ = 2.65, the powders in two tracks can be effectively coated together with minimal discernible gaps. However, melting may occur in this case, which alters the microstructure and yields a reduced hardness compared to extensively deformed material. In view of this, the multi-layer multi-track coating becomes more complex as the impact velocity should be appropriately optimized.

**Figure 26:**
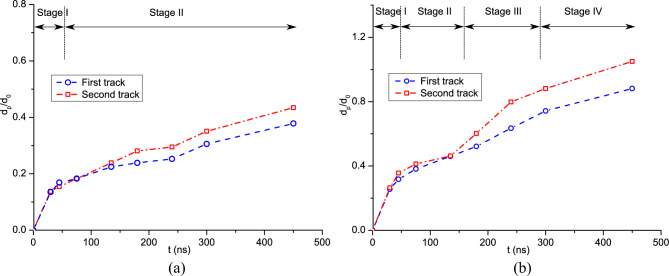
Time histories of the coating depth in the two tracks, (**a**) *V*_*d*_ = 1.47 and (**b**) *V*_*d*_ = 2.65. For the convenience of comparison, the coatings of the two tracks are set with the same starting time.

Figure 26 presents the time histories of the coating depth in the two tracks. To facilitate a comparison, we designate the starting time of coating for the second track as 0. It is observed that the second track has a deeper coating compared to the first one, particularly when subjected to a higher impact velocity of *V*_*d*_ = 2.65. As previously mentioned, the distinct four-stage variation pattern observed in CS at lower impact velocities is not discernible, and only two stages can be identified. At a higher impact velocity, the coating depths in the two tracks are relatively similar during stages I and II, with minor disparities attributable to variations in the actual powder distributions. However, the coating depth of the second track increases rapidly during stage III, while the increase slows down in the last stage. As previously discussed, a dense powder layer is yet to form during the initial two stages. In the third stage, the extrusion effect of the first track on the second track becomes obvious, leading to a deeper coating for the latter. Conversely, when the impact velocity is relatively low, the powder bed with a higher porosity is generated, where the powders in different tracks have less extrusion effects.

## Conclusions

Despite extensive numerical research on CSAM, there has been very little, if any, work focused on simulating the CSAM process in a realistic way while considering different powder sizes, morphologies, and distributions. Addressing this gap, our paper proposed an advanced parallel meshless computational scheme featuring a modified Johnson–Cook model that achieved more accurate and efficient CSAM process modeling results. The DFPM model strikes a balance between accuracy and stability and was extended to simulate an additive manufacturing operation for the first time. The integration of the modified JC model in the current method allows for the consideration of strain rate sensitivity effects during the plastic deformation of the powders. Based on comprehensive studies with numerical simulations and comparisons to experimental results, key findings can be summarized as follows:

1. The developed meshless computational framework, incorporating the modified JC model, effectively simulates powder deformations at high strain rates and yields results closer to experimental findings compared to the conventional JC model.
2. The formation of a bonded powder layer is successfully reproduced by the present simulations. The variation of coating depth in CS with different powder sizes can be divided into four stages, whereas it only exhibits a three-stage variation in CS with a uniform powder size. This discrepancy arises from the fact that uniformly distributed powders with the same size form the initial layer of bonding powders more easily, resulting in a shorter or less pronounced stage II.
3. In CS with ellipsoidal powders, the coating depth significantly decreases with an increasing length-diameter ratio *a*/*b*. Moreover, larger length-to-diameter ratios lead to increased porosity in bonding powders, so powders should be produced as close as possible to a spherical shape. Due to the challenges faced by ellipsoidal powders in forming a dense bonding layer, stage II persists for a longer duration, while stage III becomes shorter at higher impact velocities. However, at lower impact velocities, the coating depth increases slowly during the CS process, following a single-stage growth pattern.
4. The present meshless scheme can simulate multi-layer multi-track CSAM with experimentally observed powder sizes and distributions. A substantial quantity of powders reaching tens of thousands, with approximately ten million SPH particles, can be simulated by the developed meshless method. The bonding and extrusion effects between two tracks at lower and higher impact velocities are analyzed. To achieve a densely bonded powder bed while mitigating over extrusion of distinct tracks, the powder impact velocity needs to be appropriately optimized.
5. When a larger number of powders are considered, the present meshless scheme shows its significant advantages over existing ones. We have also conducted experiments for multi-layer multi-track CSAM, obtaining cross-section views of the powder track and porosity parameters. In future investigations, deeper analyses on porosity formation mechanisms in the multi-material CSAM will be conducted with comparisons to lab-scale geometries and experimental observations.

## Data Availability

The data used to support the findings of this study are available from the corresponding author upon request.
